# Photoswitchable isomers to improve grain boundary resilience and perovskite solar cells stability under light cycling

**DOI:** 10.1038/s41560-026-01993-z

**Published:** 2026-02-25

**Authors:** Zuhong Zhang, Rui Zhu, Guixiang Li, Ying Tang, Hongzhuo Wu, Jinbo Zhao, Jiaxin Wu, Thomas W. Gries, Artem Musiienko, Shengnan Zuo, Zhe Li, Bingchen He, Zhenhuang Su, Xingyu Gao, Weiwei Zuo, Bo Hou, Jorge Pascual, Luca Sapienza, Luyao Wang, Lin-Long Deng, Yu Jia, Gang Li, Paul J. Dyson, Mohammad Khaja Nazeeruddin, Michael Saliba, Antonio Abate, Meng Li

**Affiliations:** 1 Key Lab for Special Functional Materials of Ministry of Education, National and Local Joint Engineering Research Center for High-Efficiency Display and Lighting Technology, School of Nanoscience and Materials Engineering, Collaborative Innovation Center of Nano Functional Materials and Applications, Henan University, Kaifeng, People’s Republic of China; 2 School of Materials Science and Engineering, Southeast University, Nanjing, People’s Republic of China; 3 Helmholtz-Zentrum Berlin für Materialien und Energie GmbH, Berlin, Germany; 4 School of Engineering and Materials Science (SEMS), Queen Mary University of London, London, UK; 5 Shanghai Advanced Research Institute, Chinese Academy of Sciences, Shanghai, People’s Republic of China; 6 Institute for Photovoltaics (ipv), University of Stuttgart, Stuttgart, Germany; 7 School of Physics and Astronomy, Cardiff University, Cardiff, UK; 8 Polymat, University of the Basque Country UPV/EHU, Donostia-San Sebastian, Spain; 9 Department of Engineering, University of Cambridge, Cambridge, UK; 10 State Key Lab for Physical Chemistry of Solid Surfaces, Department of Chemistry, College of Chemistry and Chemical Engineering, Pen-Tung Sah Institute of Micro-Nano Science and Technology, Xiamen University, Xiamen, People’s Republic of China; 11 Department of Electrical and Electronic Engineering, Research Institute for Smart Energy (RISE), Photonic Research Institute (PRI), The Hong Kong Polytechnic University, Hong Kong, People’s Republic of China; 12 Institute of Chemical Sciences and Engineering, École Polytechnique Fédérale de Lausanne (EPFL), Lausanne, Switzerland; 13 School of Integrated Circuits, Southeast University, Wuxi, People’s Republic of China; 14 Helmholtz Young Investigator Group FRONTRUNNER, Institute of Energy and Climate Research (IEK-5)-Photovoltaics, Forschungszentrum Jülich, Jülich, Germany; 15 Department of Chemistry, Bielefeld University, Bielefeld, Germany; 16 Department of Chemical, Materials and Production Engineering, University of Naples Federico II, Naples, Naples, Italy

**Keywords:** Materials for devices, Renewable energy

## Abstract

Realizing stable and scalable perovskite solar cells (PSCs) under real-world outdoor conditions remains a challenge for deployment. Here we report a strategy to improve the resilience of the grain boundary, achieving simultaneous improvement in the power conversion efficiency and long-term operational durability under realistic light cycling and ultraviolet exposure of PSCs. By integrating photoswitchable isomers at grain boundaries, we suppress lattice bond rupture and defect accumulation during repeated light cycling through light-triggered dynamic damage release. This approach stabilizes the triple-cation lead-based perovskite lattice against photoinduced distortions and degradation pathways. As a result, the PSCs retain over 95% of their initial performance after 2,000 h of ultraviolet-containing light cycling at 65 °C and 500 thermal cycles between –40 °C and 85 °C, and deliver a power conversion efficiency of 27.2% (certified as 26.9%). Our strategy improves the operational stability and commercial viability of triple-cation perovskite photovoltaics.

## Abstract (web summary)

The operational stability of perovskite solar cells remains a challenge. Zhang et al. incorporate photoswitchable isomers at grain boundaries to improve their resilience and the stability of the solar cells under light cycling and ultraviolet exposure.

## Main

Perovskite solar cells (PSCs) have emerged as a promising technology in the photovoltaic (PV) sector, possessing high efficiency and low production costs. However, a key challenge for commercializing PSCs is related to their stability, particularly under real-world operating conditions^1^. Although recent advancements have improved PSCs against light^2,3^, heat^4,5^, bias^6,7^ and humidity^8,9^, their reliability in outdoor environments remains underdeveloped, where daily and seasonal weather conditions are highly variable.

In outdoor scenarios, PV devices primarily suffer from fluctuating temperatures and varying light intensities^1,10^. Unlike constant temperature or illumination, cyclic environmental stressors are potentially more severe accelerators of ageing, challenging the compatibility and durability of PSCs. Recently, there has been substantial progress in enhancing the temperature cycling stability of PSCs through strategies such as implementing robust polymer dipoles to buffer lattice stress and a chiral-structured heterointerface that improves mechanical reliability^11,12^.

Despite progress, the impact of light cycling remains underexplored, and when simulating outdoor conditions, the high-energy UV light could cause irreversible damage, thereby substantially reducing degrading device performance^13^. Furthermore, cyclic illumination would generate more adverse impacts and accumulate detrimental effects, ultimately shortening the lifespan and reliability of PSCs. Here the standard accelerated ageing tests, such as continuous light soaking, in replicating the outdoor cycling processes, are inadequate.

Here we employ a light-cycling protocol to mimic alternating day–night operation and show that rapid cycling accelerates PSC ageing by promoting internal-stress accumulation and grain-boundary (GB) fragmentation in the perovskite absorber. We further demonstrate a GB-resilience strategy in which photoswitchable isomers anchored at GBs reversibly switch between *trans* (*E*) and *cis* (*Z*) states, enabling dynamic regulation of local lattice strain during cycling. This reversible molecular response dissipates intergranular stress, mitigates the development of cycling-induced lattice strain and GB damage and thereby enhances operational durability under cyclic illumination, including under harsher cycling stressors. As a result, the PSCs retain over 95% of their initial performance after 2,000 h of stringent, UV-containing light cycling at 65 °C. The conclusions are supported by cycling measurements and corroborated by structural and spectroscopic evidence of strain evolution and GB integrity, together with atomistic modelling of representative GB configurations.

## Solar cell degradation under light cycling

To replicate environmental stressors, we analysed global temperature and sunlight duration data, which covers weather, location, season and other parameters, over the past five years (https://weatherspark.com/) (Supplementary Fig. 1). In these data, real-world temperature and light exposure fluctuate, typically in a cyclical pattern. This suggests that light cycling more closely reflects the operational light conditions that devices will encounter in real-world operation.

**Fig. 1:**
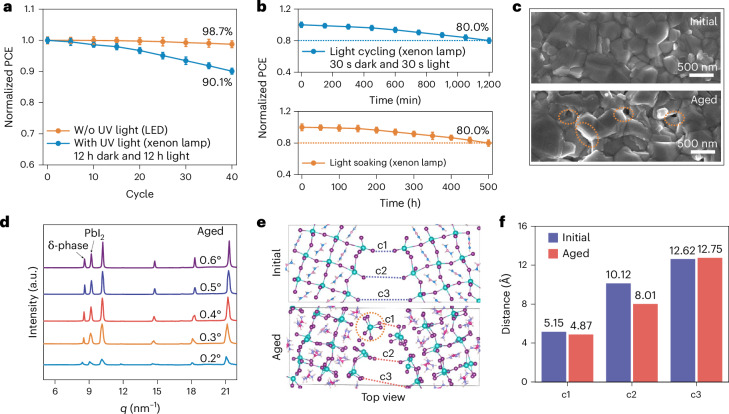
Perovskite device and film decay under UV-containing light cycling. **a**, PCE as a function of light cycling using LED or xenon lamp light (12 h light and 12 h dark). Solid lines show mean values, and error bars indicate mean ± SD. The initial average PCEs for the LED and xenon lamp ageing groups were 24.5% ± 0.26% and 25.0% ± 0.24%, respectively (*n* = 6). W/o, without. **b**, PCE as a function of rapid light cycling (30 s light and 30 s dark) and light soaking using a xenon lamp. Solid lines show mean values, and error bars indicate mean ± SD. The initial average PCEs for the light cycling and light soaking groups were 24.8% ± 0.83% and 24.4% ± 1.02%, respectively (*n* = 6).**c**, SEM images of perovskite films before and after 80 light cycles (xenon lamp). Initial and aged denote the films before and after light cycling. Orange circles mark degraded regions.**d**, GIWAXS one-dimensional integrated curves of an aged perovskite film under different incident angles (xenon lamp, 80 cycles).**e**, Top-view atomistic models of the perovskite GB before and after field-assisted ageing. The initial (top) and aged (bottom) GB configurations are shown. Pb and I atoms are shown as cyan and purple spheres, respectively. The organic ions are shown in a ball-and-stick representation (C, pink/magenta; N, blue; H, white). The dashed lines (c1–c3) mark three representative lattice sites at the GB where the interfacial spacing is evaluated (blue, initial; red, aged). Orange dashed circles highlight locally distorted/disordered regions formed after ageing.**f**, The values of c1, c2 and c3 are derived from**e**. Source data

The degradation of perovskite films and devices (device fabrication in Supplementary materials) was investigated under simulated sunlight to understand the damage caused by different lighting conditions. Following the International Summit on Organic PV Stability (ISOS) protocols, sunlight source-dependence cycling with 12 h of light and 12 h of dark (ISOS-LC-2) was conducted^14^. Under light-emitting diode (LED) cyclic illumination, the perovskite device retained 98.7% of its initial efficiency after 40 cycles, which could be attributed to dark-state healing, consistent with previous reports^1,15^ (Fig. 1a). However, when using a solar simulator that included UV light, the devices exhibited a faster performance decay, retaining 90.1% of the initial efficiency, even after the same dark-state recovery. Thus, the greater damage highlights the risk posed by UV-containing light cycling for long-term durability. We sought to establish an accelerated light cycle that approximates light soaking under maximum power point tracking (MPPT) by shortening the dark-state recovery time. We observed accelerated degradation of the devices under rapid light cycling (Supplementary Fig. 2). Specifically, PSCs subjected to 30-s-light–30-s-dark cycles for 1,200 cycles exhibited power conversion efficiency (PCE) loss equivalent to that caused by 500-h 1-sun MPPT (Fig. 1b). Notably, this light-cycling period took only 1,200 min (20 h), dramatically reducing the light-induced ageing time.

Following light cycle-induced damage, the perovskite film showed evident morphological changes, for example, increased pinholes at the GBs (results from scanning electron microscopy (SEM) images in Fig. 1c). Through grazing-incidence wide-angle X-ray scattering (GIWAXS) analysis, a substantial increase in the PbI_2_ peak and the formation of the δ phase was observed (Fig. 1d and Supplementary Fig. 3), which reduces the photoactivity of the perovskite film^16,17^. We further demonstrate that the performance degradation mainly originates from the perovskite layer by comparing the performance of devices with different self-assembled monolayers (SAMs) before and after UV light ageing (Supplementary Fig. 4). To understand the reasons for the degradation of the perovskite film, we calculated the dynamic deformation of perovskite crystal grains with an ab initio molecular dynamics method. The Σ5(130) was identified as the most stable configuration and thus adopted as the perovskite crystal model, as it shows the minimum GB energy (Supplementary Fig. 5 and Supplementary Table 1). The representatives, c1, c2 and c3, in a GB point (top view) (Supplementary Fig. 6), shifted from 5.15, 10.12 and 12.62 Å to 4.87, 8.01 and 12.75 Å, and PbI_2_ was generated in the aged structure (Fig. 1e,f and Supplementary Figs. 7 and 8). The short-distance shift caused by the perovskite’s composition diffusion revealed that a cycled energy field induces stress accumulation within the perovskite crystal, which results in unfavourable phase transitions and crystal defects such as bond breakage, leading to the degradation of the perovskite film. The Pb 4*f* X-ray photoelectron spectroscopy (XPS) confirmed the presence of Pb^0^ (Supplementary Fig. 9), indicating that UV-inclusive solar illumination causes perovskite to decompose into methylammonium iodide (MAI), formamidinium iodide (FAI) and PbI_2_, while the PbI_2_ further decomposes into Pb^0^ and I_2_ (refs. ^18,19^). It would appear that full-spectrum solar light cycling induces permanent changes in the absorber that could critically impact device durability. Provided by these analyses, we propose that light cycling could induce interfacial stretching between grains that leads to perovskite degradation into PbI_2_ and partial ion loss, leaving vacancies at the GBs, accelerating device damage during continuous cycling^20^ (Supplementary Fig. 10).

## Enhancing light-cyclic resilience of perovskite films

We considered that a GB inverse thermal expansion-driven film strain-release coupling strategy might reduce dynamic perovskite damage induced by cyclic illumination. An additive (4-(phenylazo)benzoic acid, Ca-Abz) that undergoes a UV light-induced (reversible) isomerization was incorporated into the structure of triple-cation Pb perovskite with a composition of Cs_0.05_MA_0.05_FA_0.9_PbI_3_ (ref. ^21^).

**Fig. 2:**
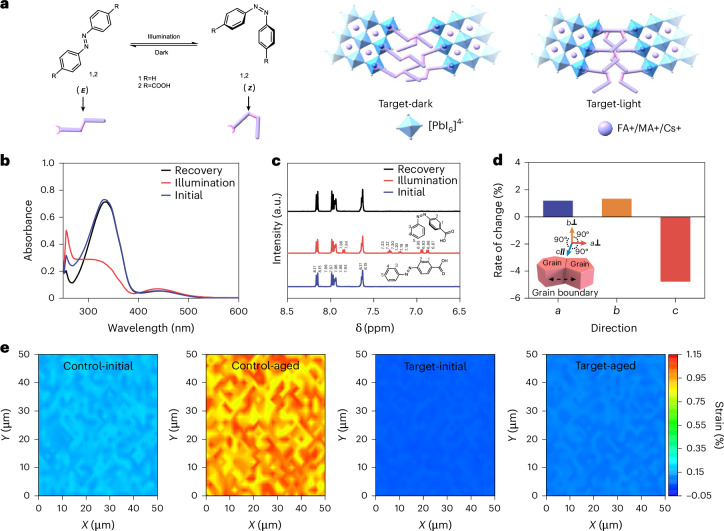
Dynamic resilience strategy employing a photoswitchable isomeric compound. **a**, Reversible photoisomerization of azobenzene derivatives and the corresponding grain-boundary configurations. Left: Azobenzene derivatives (1: R = H; 2: R = COOH) undergo reversible *E* → *Z* isomerization under illumination and relax back to the *E* form in the dark. Right: at perovskite grain boundaries, the *E* isomers adopt a more linear bridging configuration between neighbouring [PbI_6_]^4−^ octahedra (Target-dark), whereas light-induced *Z* isomers exhibit a bent conformation (Target-light). **b**,**c**, UV–vis absorption spectra (**b**) and ^1^H NMR spectra of Ca-Abz during reversible photoisomerization (**c**). Initial (blue), illuminated (red) and recovered (black) states are shown. Structures indicate the *E* and *Z* forms. Numbers denote assigned protons corresponding to the labelled resonances. **d**, Rate of change in *a*, *b* and *c* direction, where *a*, *b* and *c* represent the three-dimensional direction of grains, which is shown in the inset images. **e**, Confocal micro-Raman spectroscopy mapping images of perovskite films, which were obtained from the Raman peak.

In the dark, Ca-Abz predominantly adopts the planar *E* form, stabilized by both its intrinsic molecular orbital configuration and interfacial bonding with the perovskite GB (Fig. 2a). In contrast, the *Z*-form is twisted due to steric repulsion between adjacent rings, disrupting π-conjugation and increasing the molecular orbital energy. Upon UV illumination, n(N) electrons are excited to the π*(N = N) antibonding orbital, weakening the N = N bond and lowering the rotational barrier, thereby facilitating *E* → *Z* photoisomerization. This is evidenced by the spectral evolution: the π–π* band at ≈350 nm decreases while a new n–π* band emerges at ≈450 nm (Fig. 2b). The process is reversible, as the *E* form spectra recover after dark storage, consistent with spontaneous thermal back-isomerization. Reversible ^1^H nuclear magnetic resonance (^1^H NMR) spectroscopy splitting further confirms this transformation (Fig. 2c). Single-molecule transition-state (TS) analyses indicate a 2.16 eV barrier, confirming that the transition is feasible under UV light (>3.1 eV; Supplementary Fig. 11).

Collectively, this reversible *E⇄Z* transformation allows Ca-Abz to dynamically respond to lattice strain, effectively mimicking the perovskite contraction and expansion during light cycling and acting as a buffer (Supplementary Fig. 12). Notably, Ca-Abz has a carboxylic acid group at one end, which can anchor to the perovskite grain interface, passivating it, while the other end remains flexible, thus forming an ordered buffer in the GBs. We also compared Ca-Abz with azobenzene (Abz) and azobenzene-4,4’-dicarboxylic acid (Dca-Abz), which feature different numbers of functional groups on the benzene rings (Supplementary Fig. 13). Among the isomer additives tested, Ca-Abz resulted in the best light-cycling stability (Supplementary Fig. 14), attributed to its single anchoring group (C = O), which facilitates ordered isomerism. In contrast, Abz, lacking an anchoring group, tends to be disordered due to the absence of an effective passivation site, while the two anchoring groups in Dca-Abz limit its photoswitchable capacity due to restricted molecular flexibility. We also compared the photoswitchability of pure Dca-Abz and the Dca-Abz mixture with PbI_2_ by UV–vis spectra. As shown in Supplementary Fig. 15, pure Dca-Abz shows more obvious *trans* structure phototransfer to *cis* structure than Dca-Abz/PbI_2_, meaning that the interaction between the two-site C = O in Dca-Abz and Pb^2+^ limits photoswitchability. Extracting from these results, we could support the advantage of the target molecule used in perovskite devices under light cycling. To explore which isomer stably exists at 85 °C, we measured the Ca-Abz at 85 °C under light and dark conditions. Consistent with our hypothesis, the *trans* structure mainly exists at dark 85 °C, and the *cis* and *trans* structures exist at light 85 °C (Supplementary Fig. 16). This result indicates that the isomer is stable at 85 °C, where the molecule could normally work at elevated temperature.

Upon the Ca-Abz functionalization, we observed negligible changes in the morphology and crystal structure of the Ca-Abz-treated perovskite films under light cycling, as demonstrated by SEM and GIWAXS measurement (Supplementary Figs. 17–19). XPS spectra also identified that Ca-Abz stabilized the perovskite film from light ageing (Supplementary Fig. 20). We further investigated the mechanism behind Ca-Abz stabilizing the perovskite structure. To investigate the molecule’s optimization on GBs, we investigated the unit cell structure using density functional theory and first principles molecular dynamics, modelling the *c* axis along the GB. The change rates of lattice parameters in the perovskite GB were 1.18% (*a* axis), 1.32% (*b* axis) and –4.77% (*c* axis) as Ca-Abz transitions from the *E* isomer to the *Z* isomer (Fig. 2d and Supplementary Figs. 21–23). Notably, the lattice variation along the *c* axis suggests that molecular switching in Ca-Abz modulates interfacial strain and coupling at the perovskite GB, revealing an association between molecular conformation and lattice structure. This buffering effect would reduce stress and suppress perovskite decay. The rate of change of the lattice parameters along the *c* axis in the perovskite GBs with Abz and Dca-Abz also verify that Ca-Abz effectively mitigates lattice damage (Supplementary Fig. 24). For clarification, the adsorption energies of Ca-Abz on the perovskite surface were calculated for both *E* and *Z* configurations. The *Z* isomer (−11.01 eV) binds slightly more strongly to the surface than the *E* isomer (−10.99 eV), suggesting a marginally higher surface affinity. This suggests that light-induced isomerization (*E* → *Z*) favours enhanced molecular passivation and improved structural stability of the perovskite, which is consistent with our experimental observations. The strain in the perovskite films was evaluated by confocal micro-Raman spectroscopy mapping. The strain changes were mainly reflected in the changes in the length of Pb–I bonds obtained through the Raman peak shift^22^. As shown in Fig. 2e, in a 50 × 50 μm^2^ area, the average strains of control-initial (unmodified), control-aged, target-initial (processed with Ca-Abz) and target-aged films were 0.17, 0.95, 0.06 and 0.10%, respectively, showing that the strain in target films was largely suppressed. This was attributed to the Ca-Abz *E⇄Z* of Ca-Abz switching at the perovskite GBs during light cycling. Grazing-incidence X-ray diffraction was also used to investigate film strain during light cycling, with both techniques confirming the release of strain in the target film (Supplementary Fig. 25). As shown in Supplementary Figs. 26–28, the target films exhibit lower in-plane tensile stress and out-of-plane compressive stress than the control films, both before and after ageing. These quantitative indicators support our conclusion that the structural modification effectively relieves stress and suppresses degradation. As shown in Supplementary Fig. 29, devices without Ca-Abz exhibited a notable increase in trap density of states after light cycling, whereas Ca-Abz-modified devices maintained low and stable defect levels. Time-of-flight secondary-ion mass spectroscopy analysis (Supplementary Fig. 30) further confirms that Ca-Abz effectively suppresses I^−^ ion migration, leading to enhanced operational stability by mitigating light-cycling-induced lattice stress and defect formation.

## Improved charge dynamics and PV performance

**Fig. 3:**
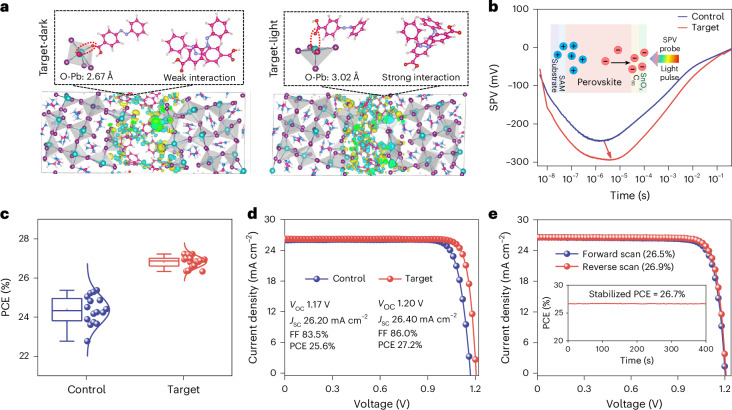
PV performance of the PSCs. **a**, Charge-density-difference isosurfaces of Ca-Abz at the perovskite grain boundary in the *E* (Target-dark) and *Z* (Target-light) configurations. Yellow and cyan indicate electron accumulation and depletion, respectively. The O–Pb interaction site is highlighted by the red dashed ellipse. Atom colours: Pb (cyan), I (purple), C (pink), N (blue), O (red) and H (white). **b**, trSPV measurement of devices with the structure: substrate/SAM/perovskite/C_60_/SnO_*x*_. The arrow indicates the more negative SPV minimum of the Target device relative to the Control. **c**, Statistics of PCEs from 16 independent devices. Box plots show median (centre line), mean (open square), 25th and 75th percentiles (box bounds) and minima/maxima within 1.5× interquartile range (whiskers). **d**, *J*–*V* curves of champion control and target devices under AM1.5 G. **e**, Certified *J*–*V* curves and stabilized PCE from the National PV Industry Measurement and Testing Centre. Credit: **e**, NPVM. Source data

To evaluate the impact of Ca-Abz on the photophysical properties of the perovskite film, we comprehensively analysed the crystal structure and optoelectronic characteristics of the perovskite films under periodic illumination. According to the shifted stretching vibrations of C–O and C = O (Fourier-transform infrared (FTIR) spectra in Supplementary Fig. 31) and Pb 4*f* (XPS spectra in Supplementary Fig. 32), the carboxylic acid group in Ca-Abz interacts with Pb^2+^ ions in the perovskite, presumably as a carboxylate via loss of the proton^23–26^. This type of coordination bond passivates perovskite defects. We carried out GIWAXS to observe the crystallinity of perovskite films across different depths (Supplementary Fig. 33) by changing the incident angles (0.2, 0.4 and 0.6 degrees). Compared to the control perovskite film, the target films displayed a more uniform (100) crystal orientation and higher crystallinity intensity at *q* ≈ 10 nm^−1^, indicating enhanced perovskite film quality and consequently enhanced optoelectronic performance. We performed charge difference calculations to analyse the interaction between Ca-Abz and the perovskite, as shown in Fig. 3a. In the *E* isomer, the molecule forms a O–Pb bond with a distance of 2.67 Å and exhibits additional π–π interactions between the benzene rings. Upon light-induced *E* → *Z* isomerization, the O–Pb distance increases to 3.02 Å and intermolecular interactions are strengthened. Quantitative analysis of the charge distribution reveals that –7.51 |e| are transferred from the perovskite to the molecule in the *E* configuration, increasing to –7.82 |e| in the *Z* configuration, indicating stronger coupling and enhanced charge redistribution at the interface. These changes suggest that the light-driven *E* → *Z* transition promotes charge accumulation and depletion across the GB, facilitating carrier transport and suppressing interfacial nonradiative recombination.

We further investigated the charge extraction dynamics using transient surface photovoltage (trSPV) measurements (Fig. 3b and Supplementary Fig. 34). The inset shows the trSPV response of the perovskite at an excitation wavelength of 688 nm. The control sample exhibited a negative SPV signal, indicating free electrons dominating near the top surface. In contrast, the Ca-Abz-treated sample showed a larger SPV signal, corresponding to enhanced electron extraction that is beneficial to device performance^27,28^. Photoluminescence (PL) and time-resolved photoluminescence (TRPL) spectra further supported the improved film quality (Supplementary Figs. 35 and 36 and Supplementary Table 2), as evidenced by high PL intensity and prolonged carrier lifetimes^29^. Additionally, open-circuit voltage (*V*_OC_) dependence on the light intensity, transient photovoltage (TPV) and transient photocurrent (TPC) measurements, all verified reduced defect density within devices^30–32^, which correlates with the observed improvements in charge carrier dynamics (Supplementary Figs. 37–39).

Devices based on the p–i–n architecture were fabricated with the composition fluorine-doped tin oxide (FTO) substrate/SAM/triple-cation perovskite/interface/fullerene (C_60_)/SnO_*x*_/Ag (Supplementary Fig. 40). The target devices (1 mg ml^−1^ Ca-Abz modified devices) showed an average PCE of 26.8%, substantially higher than that of the control devices, demonstrated at approximately 24.4% (Fig. 3c and Supplementary Fig. 41). The PCE of the champion target device is 27.2%, compared to 25.6% for the champion control device. This improvement primarily stems from increases in both open-circuit voltage (*V*_OC_, from 1.17 to 1.20 V) and fill factor (FF, from 83.5 to 86.0%) (Fig. 3d and Supplementary Fig. 42). The target device delivered a stabilized PCE of 27.0%, considerably higher than the control device (24.7%) (Supplementary Fig. 43). The integrated short-circuit current density (*J*_SC_) obtained from external quantum efficiency (EQE) measurements is 26.15 mA cm^−^^2^ (Supplementary Fig. 44), consistent with the *J*–*V* value. The bandgap of the perovskite was determined to be 1.53 eV by the derivative of EQE for wavelength (Supplementary Fig. 45). The target device was certified at the National PV Industry Measurement and Testing Center with a PCE of 26.9% (Supplementary Figs. 46). A PCE of 26.7% that was stabilized after 400 s MPPT was also certified under an aperture area of 7.82 mm^2^ (Fig. 3e and Supplementary Fig. 47).

## Operational stability of the devices

**Fig. 4:**
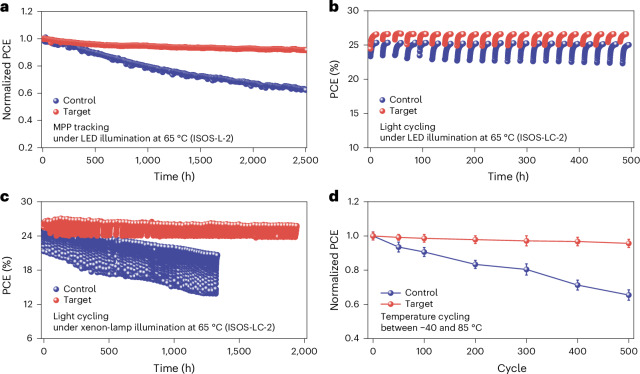
Ageing testing of perovskite solar cells. **a**, MPPT for unencapsulated devices in N_2_ under LED-simulated 1-sun illumination. The initial PCEs for the control and target ageing groups were 25.2% and 26.8%, respectively. **b**, Light cycling under the LED light illumination (12 h of light and 12 h of dark). **c**, Light cycling under the solar simulator (12 h of light and 12 h of dark). **d**, Rapid temperature cycling ageing between −40 and 85 °C. Solid lines show mean values, and error bars indicate mean ± SD. The initial average PCEs for the control and target ageing groups were 24.8% ± 0.58% and 26.5% ± 0.60%, respectively (*n* = 6). Source data

Subsequently, we evaluated the operational stability of our PSCs using the International Summit on Organic PV Stability (ISOS)-L-2 protocol^14^. Figure 4a shows that under continuous one-sun MPPT, the target PSC retained 91.7% of its initial PCE after 2,500 h, whereas the control PSC retained only 62.3%.

PV performance of the devices was further evaluated under two types of light cycling, that is, LED and xenon lamp illumination. According to the ISOS light-cycling protocol (ISOS-LC-2), we employed 24-h cycles (12 h light and 12 h dark) to mimic the daily sun cycle at a fixed temperature of 65 °C. Under LED light cycling, the target device retained almost 100% of its initial efficiency after 500 h, whereas the control devices degraded by about 1.5% after the same exposure time (Fig. 4b). Under xenon lamp cycling, the target device maintained stable output for nearly 2,000 h. In contrast, the control device exhibited linear degradation, with about 20.0% initial efficiency loss after 1,300 h (7.0% loss after 500 h) and large fluctuations in low-PCE regions (Fig. 4c). We attribute the more greater degradation under xenon lamp cycling compared to LED to the impact of UV light. The improved stability of the target device suggests that the dynamic isomerization process of Ca-Abz mitigates damage to UV and light cycling. Besides, light-cycling tests on MA-free Cs_0.05_FA_0.95_PbI_3_-based devices show that Ca-Abz modification effectively suppresses performance decay (∼100% retention after 600 h versus 94% for control) (Supplementary Fig. 48). SEM and GIWAXS reveal PbI_2_ formation only in unmodified films (Supplementary Figs. 49 and 50), confirming the universal stabilizing effect of Ca-Abz. To further verify the effectiveness of the Ca-Abz-containing device, we performed rapid temperature cycling of devices between –40 and 85 °C. As shown in Fig. 4d, the control devices maintained 65.5% of the initial PCE after 500 cycles, whereas the target device retained 95.7% of the initial PCE.

## Conclusions

In conclusion, continuous light cycling causes the rapid degradation of PSCs, and incorporating the photoswitchable compound efficiently stabilizes perovskite devices under cycled illumination. Light cycling leads to the accumulation of internal stress in perovskite films, leading to pronounced expansion in crystal parameters, especially at the GBs of perovskite films, which results in film degradation and directly diminishes their optoelectronic properties. By introducing photoactive isomerizable compounds at the GBs, we effectively passivate dynamical crystal defects and enhance charge separation and extraction. The target devices described here that incorporate Ca-Abz as the photoswitchable compound achieve a high efficiency of 27.2% and a certified efficiency of 26.9% (certified MPPT efficiency of 26.7%). We found that such isomers efficiently buffer film strain through a reversible structural transformation triggered by UV-containing light, enabling the high-efficiency devices to maintain stable operation under prolonged light cycling. This approach not only converts high-energy UV light to prevent damage but also enhances the durability of PV devices during light cycling. We believe that the dynamic regulation using photoswitchable isomeric compounds marks an advancement towards the practical application of perovskite PVs in real-world scenarios.

## Methods

### Materials

FAI, MAI, methylammonium chloride (MACl) and propane-1,3-diammonium iodide (PDAI_2_) were purchased from Greatcell Solar Materials. Lead iodide (PbI_2_) and fullerene (C_60_) were purchased from Libra Technology Corporation. 3-(methylthio)propylamine hydroiodide (3-MTPAI) was purchased from Xi’an Yuri Solar Co. Ltd. 2-(9H-carbazol-9-yl)ethyl]phosphonic acid (2PACz), 4-(3,6-dimethyl-9H-carbazol-9-yl)butyl]phosphonic acid (Me-4PACz) and caesium iodide (CsI) were purchased from TCI. Organic solvents were purchased from Alfa Aesar, including dimethyl sulfoxide (DMSO, 99.8%) and *N*, *N*-dimethylformamide (DMF, 99.8%). Chlorobenzene (CB, 99.5%), isopropanol (IPA, 99.7%) and absolute ethyl alcohol (99.7%) were purchased from J&K Scientific. Azobenzene, 4-(Phenylazo)benzoic acid and azobenzene-4,4’-dicarboxylic acid were purchased from Aladdin.

### Device fabrication

#### Solution preparation

For the control perovskite solution, 1.7 mmol Cs_0.05_MA_0.05_FA_0.9_PbI_3_ (22.1 mg CsI, 13.6 mg MAI, 263.2 mg FAI and 806.8 mg PbI_2_) and 10 mg MACl were dissolved using 1 ml mixed DMF and DMSO solvents (4:1 v/v), and the solution was stirred at 60 °C for 4 h. The solution was filtered through a 0.22-μm hydrophobic PTFE filter before use. The target solution was prepared by adding 0.5, 1, 1.5 and 2 mg of 4-(Phenylazo)benzoic acid to the control perovskite solution, and the other steps were the same as those for the control solution. The self-assembly layer (SAM, 0.17 mg 2PACz, and 0.33 mg Me-4PACz in 1 ml absolute ethyl alcohol) and 3-MTPAI (1.5 mg ml^−1^) and PDAI_2_ (0.5 mg ml^−1^) mixed solutions in IPA were stirred at 25 °C for 12 h before use and did not need to be filtered.

#### Device preparation

Fluorine-doped tin oxide (FTO) glasses were cleaned using detergent, then sonicated in deionized water (10 min), acetone (10 min) and IPA (10 min), respectively. After sonically cleaning, the FTO glasses were dried using a flow of N_2_ gas. To remove any remaining organic contaminants on the surface and to enhance wettability, the cleaned FTO glasses were treated with UV-ozone for 30 min and were transferred to the glovebox. The SAM was prepared by spin-coating 100 μl mixed SAM solution at 3,000 rpm for 30 s and annealed at 100 °C for 10 min. The perovskite precursor solution (100 μl) was spin coated at 1,000 rpm for 10 s and 5,000 rpm for 30 s, and the 200 μl CB was dropped at 25 s and annealed at 100 °C for 45 min. 3-MTPAI and PDAI_2_ mixed solutions (100 μl) were spin coated on the perovskite film at 4,000 rpm for 30 s and annealed at 100 °C for 5 min. After that, a 40 nm layer of C_60_ (0.03-0.05 nm s^−1^), a 20 nm layer of SnO_*x*_ and a 100 nm layer of Ag (1–2 nm s^−1^) were sequentially evaporated using a metal mask. The SnO_*x*_ was deposited by thermal atomic layer deposition. The C_60_ and Ag were thermally deposited using vacuum evaporation.

### Characterization

#### Device performance

The films were prepared by spin coater purchasing from Jiangsu LEBO Scientific Instrument Co. Ltd. *J*–*V* curves were measured under AM 1.5 G illumination from a solar simulator (EnliTech, SS-X50, with A+ spectrum) and a Keithley 2400 source meter; the light intensity was calibrated through a reference silicon solar cell obtained from National Institute of Standards and Technology (America). The *J*–*V* curves were measured for both forward (−0.1 to 1.22 V) and reverse (1.22 to −0.1 V) scans. The scan rate was 20 mV s^−1^ with a dwell time of 10 ms per point. The *J*–*V* curves were scanned with a 9.82 mm^2^ metal aperture area in a glovebox. The integrated *J*_SC_ of external quantum efficiency (EQE) was obtained using the solar-cell-spectra-response measurement system (QE-R, EnliTech). TPC and TPV were measured through a transient photocurrent/voltage tester (Shanghai Jinzhu Technology Co., laser 570 nm). The stability measurements were carried out under LED or xenon lamp light with an intensity of 100 mW cm^−^^2^. The intensity was calibrated with a standard silicon solar cell, and all devices were measured in an inert atmosphere in a glovebox. The xenon lamp spectrum used for stability measurements was obtained from the manufacturer-provided spectral report (Supplementary Fig. 51), and the emission spectrum of the white-light LED source is shown in Supplementary Fig. 52. Temperature regulation is achieved using a standalone controller (mK2000B) integrated with a hot/cold stage (HCP421-PM), a 100 Ω platinum resistance temperature detector sensor, a water circulation system, a liquid nitrogen cooling module (INSTEC LN2-P), a KF16 vacuum port, an LN2 dewar, a DC power source and the Instec App for software-based management. The system offers a temperature range from −190 to 400 °C, maintaining stability within ±0.05 °C. Heating and cooling rates range from a minimum of ±0.1 °C h^−1^ to a maximum of +150 °C min^−1^ and −50 °C min^−1^ at 37 °C, respectively, with a temperature control resolution of 0.001 °C. During thermal cycling tests, the sample is housed within a chamber that allows precise vacuum and temperature control. The device is cycled between −40 °C and +85 °C, with liquid nitrogen serving as the primary coolant. Proportional–integral–derivative (PID) settings are used to fine tune the thermal control parameters.

#### Transient surface photovoltage measurements

Before the trSPV measurements, all samples were encapsulated with a 170-μm cover glass and UV-curable glue (BluFixx MGS) to avoid exposure to humidity and oxygen. The measurements were conducted on a set-up built in house. Excitation succeeded from the top surface, exposing either perovskite or C_60_, at eight different photon energies (1.2, 1.4, 1.6, 1.8, 2.0 and 2.2 eV) from a tunable pumped pulse Nd: YAG laser (EKSPLA, NT230-50-SH/SF-SCU-2H) at 3–6 ns pulse time and 2 Hz frequency. Laser fluence was controlled via neutral density filters to around 15.0 nJ cm^−2^ for 1-sun equivalent. The final transient is an average of 20 curves recorded in total. The transients were measured with an oscilloscope card (Gage, CSE 1622- 4GS, 200 MS s^−1^) using software for logarithmic read out developed in house.

#### Perovskite film properties

PL spectra were recorded on a time-resolved variable-temperature fluorescence spectrometer (Edinburgh Instruments FLS1000) using a xenon lamp as the excitation source. TRPL measurements were performed on the same system using 460 nm pulsed excitation at a repetition rate of 100 kHz. The excitation spot diameter was ~1 mm. The excitation fluence was 40 nJ cm^−2^ per pulse (0.04 µJ cm^−2^), corresponding to an average power density of ~4 mW cm^−2^. PL decays were monitored at 810 nm (near the emission peak). A cooled red-sensitive photomultiplier tube was used as the detector. ^1^H NMR spectra were recorded on a 400 M NMR spectrometer (Bruker, 400 M NMR spectrometer). FTIR spectra were recorded on a Bruker VERTEX 70 spectrometer. XPS was performed on a Shimadzu, AXIS, SUPRA+ instrument. Field emission scanning electron microscopy images were obtained on a JEOL JSM-7610F Plus instrument. The X-ray diffraction patterns of perovskite films measured at different incident angles were recorded on a grazing-incidence X-ray diffraction (Rigaku SmartLab 3 kW). GIWAXS was carried out on the BL03HB beamline at the Shanghai Synchrotron Radiation Facility; the incident wavelength was 1.2398 Å and the energy was 10 keV. The one-dimensional spectra and two-dimensional patterns were obtained through FIT2D and GIWAXS-tools software. All dates were calibrated using a Lanthanum hexaboride reference sample. Raman mapping was measured on a laser Raman microscope system (RAMAN-11, Nanophoton). The excitation laser wavelength was 532 nm, with an excitation density of 26.7 W cm^−^^2^ (laser current at 100%), grating at 600 g mm^−1^ and a neutral density filter at 0.91%. The readout speed was 2 MHz (high grain, low readout port and slit width of 50 mm). The type of objective lens was ×50/0.45. The CCD temperature is –69 °C. The detection range was 50 μm × 50 μm^2^ (delta was 2,000 nm, exposure time of 1 s, averaging time of once). The wavenumber shift compensation was 5 cm^−1^. The relationship between the Raman wavenumber of the Pb–I bond (*ω*_Pb-I_) and lattice constant *d*_(100)_ is *d*_(100)_ = 0.00892*ω*_Pb-I_ + 5.1105, where *ω*_Pb-I_ can be obtained by Gauss fitting the Raman data (*R*^2^ > 0.98). The strain was calculated using the equation: *ε* = (*d* − *d*_0_)/*d*_0_(ref.^22^).

### Theoretical calculation

A Σ5(130) of cubic FAPbI_3_ was constructed following the procedure reported previously^20^. The structure was first optimized under finite-temperature conditions to obtain a stable configuration, which was then used as the initial structure for constant-number, constant-volume and constant-temperature (NVT) molecular dynamics (MD) simulations at 233 K. Subsequently, the GB was simulated under a temperature ramp from 233 K to 353 K in the NPT ensemble, explicitly accounting for temperature-induced energy fluctuations and thermal effects on lattice parameters. From these simulations, the temperature dependence of the lattice parameters was obtained. The resulting temperature-relaxed MD structures explicitly include finite-temperature effects and sample dynamic conformations at the GB, providing a realistic representation of molecular configurations relevant for assessing interface behaviour.

For Ca-Abz, both*E* and*Z* isomers were fully optimized as single molecules and frequency analyses were performed to confirm the absence of imaginary vibrational modes, ensuring that all optimized structures correspond to true minima on the potential energy surface. Considering the complex spatial configuration of the FAPbI_3_ GB, six adsorption configurations of Ca-Abz molecules were evaluated at the perovskite interface. These perovskite/Ca-Abz interfaces were first optimized under finite-temperature conditions, followed by NVT simulations at 233 K and NPT simulations to account for temperature-dependent lattice deformations. This approach allows sampling of the stable dynamic configurations of Ca-Abz at GBs and evaluation of their interactions with the perovskite lattice.

All MD calculations were performed using the CP2K software package with the GGA-PBE functional^33–35^. DZVP-MOLOPT-SR-GTH basis sets and Goedecker–Teter–Hutter pseudopotentials were employed^36,37^. Van der Waals interactions were treated using the vdW-DF3 functional^38,39^, as they play a critical role in interface stability. Valence wave functions were expanded in-plane waves with a cut-off energy of 400 eV. In NVT simulations, the Nosé–Hoover thermostat was applied at 233 K, with a time step of 1 fs and a total simulation time of 2,000 fs (ref. ^40^). In NPT simulations, temperature and pressure were maintained at 300 K and 1 bar using the Nosé–Hoover thermostat and barostat.

Single-molecule optimizations of Ca-Abz were carried out using the Gaussian16 software package with the B3LYP functional and 6-31 + G(d,p) basis set^41^. These calculations provide insight into the electronic structures and relative stabilities of the *E* and *Z* isomers, complementing the MD simulations at the interface.

We note that Nudged Elastic Band, which could provide quantitative activation barriers for *E* → *Z* photoisomerization, is not feasible for the perovskite/Ca-Abz system due to its large size (up to 594 atoms, 12,799.3 Å^3^, requiring ~1.7 × 10^6^ plane-wave basis functions). Instead, we conducted single-molecule TS analysis. We calculated the activation barrier for the *E*⇄*Z* transformation and performed intrinsic reaction coordinate analysis to confirm the relaxation paths towards both configurations. High-level coupled-cluster singles and doubles with perturbative triples calculations yielded a reaction energy profile. Overall, the experimentally verified reversibility, MD simulations at the perovskite interface and TS-level molecular reaction pathway collectively support the reliable and repeatable *E*⇄*Z* photoisomerization of Ca-Abz and its functional role in strain modulation at grain boundaries.

### Equations for calculation

#### Ideal factor calculation

The ideal factors were obtained by *V*_OC_ versus light intensity measurement, which can be described as follows:

1 $${V}_{\mathrm{OC}}=\frac{n{K}_{{\rm{B}}}T}{q}\mathrm{ln}I$$

where *V*_OC_ is the open-circuit voltage at different light intensities, *n*, *K*_B_, *T* and *q* are the ideal factors, the Boltzmann constant, the temperature and the elementary charge, respectively. *I* is the light intensity^29^.

#### TRPL lifetime calculation

The lifetime was fitted by the bi-exponential equation and the average lifetime was obtained from:

2 $${\tau }_{\mathrm{ave}}=\frac{{A}_{1}{{\tau }_{1}}^{2}+{A}_{2}{{\tau }_{2}}^{2}}{{A}_{1}{\tau }_{1}+{A}_{2}{\tau }_{2}}$$

Where *τ*_1_ and *τ*_2_ are the decay times of trap-mediated recombination and radiative recombination, respectively, and *A*_1_ and *A*_2_ are the amplitudes^42^.

#### Lattice strain calculation from GIWAXS

According to the 2*θ*-sin^2^*φ* method, Bragg’s Law and generalized Hooke’s Law, we can obtain the stress index σ as equation (3)^43,44^:

3 $$\sigma =\frac{E}{\left(1+v\right){\sin }^{2}\varphi }\left(\frac{{d}_{\varphi }-{d}_{n}}{{d}_{n}}\right)$$

where the *φ* and *n* are the scattering vector angles of the perovskite film surface normal direction.

The direction of *φ* and *n* are out of plane (90°, ⊥) and in-plane (0°, ∥); we can write equation (3) as equation (4):

4 $$\sigma =-\frac{E}{\left(1+v\right)}\left(\frac{{d}_{\perp }-{d}_{\parallel }}{{d}_{\parallel }}\right)$$

We adopt the convention that tensile in-plane stress is positive. Because, under our GIWAXS geometry, the sign of *σ* from equation (3) is opposite to this convention, a minus sign is included in equation (4) so that σ > 0 and σ < 0 correspond to tensile and compressive in-plane stress, respectively. Equation (4) can be written as equation (5) as *q* = 2π/*d*:

5 $$\sigma =-\frac{E}{\left(1+{{v}}\right)}\left(\frac{{{q}_{\parallel }-q}_{\perp }}{{{{q}}}_{\perp }}\right)$$

where *E* and *v* are Young’s modulus and Poisson’s ratio of the perovskite film, respectively. *E* and *v* were evaluated as 10 GPa and 0.3, respectively^45–47^.

### Reporting summary

Further information on research design is available in the Nature Portfolio Reporting Summary linked to this article.

## Supplementary information

Supplementary Information Supplementary Notes 1–3, Figs. 1–52, Tables 1 and 2 and ref. 1.

Reporting Summary

Supplementary Data Data for Supplementary Figs. 2, 4a,b, 14, 41 and 42.

## Source data

Source Data Fig. 1 Source data for Fig. 1a,b.

Source Data Fig. 3 Source data for Fig. 3c–e.

Source Data Fig. 4 Source data for Fig. 4a,d.

## Data Availability

The data that support the findings of this study are available within the Article and its Supplementary Information. Source data are provided with this paper.

## References

[1] Khenkin, M. et al. Light cycling as a key to understanding the outdoor behaviour of perovskite solar cells. *Energy Environ. Sci.* **17**, 602–610 (2024).

[2] Lin, Y.-H. et al. Bandgap-universal passivation enables stable perovskite solar cells with low photovoltage loss. *Science* **384**, 767–775 (2024). 38753792 10.1126/science.ado2302

[3] Chen, Y. et al. Nuclei engineering for even halide distribution in stable perovskite/silicon tandem solar cells. *Science* **385**, 554–560 (2024). 39088618 10.1126/science.ado9104

[4] Tang, H. et al. Reinforcing self-assembly of hole transport molecules for stable inverted perovskite solar cells. *Science* **383**, 1236–1240 (2024). 38484063 10.1126/science.adj9602

[5] Zhao, X. et al. Operationally stable perovskite solar modules enabled by vapor-phase fluoride treatment. *Science* **385**, 433–438 (2024). 39052792 10.1126/science.adn9453

[6] Ren, X. et al. Mobile iodides capture for highly photolysis-and reverse-bias-stable perovskite solar cells. *Nat. Mater.* **23**, 810–817 (2024). 38684883 10.1038/s41563-024-01876-2

[7] Jiang, F. et al. Improved reverse bias stability in p–i–n perovskite solar cells with optimized hole transport materials and less reactive electrodes. *Nat. Energy* **9**, 1275–1284 (2024).

[8] Zou, Y. et al. A crystal capping layer for formation of black-phase FAPbI_3_ perovskite in humid air. *Science* **385**, 161–167 (2024). 38991067 10.1126/science.adn9646

[9] Sidhik, S. et al. Two-dimensional perovskite templates for durable, efficient formamidinium perovskite solar cells. *Science* **384**, 1227–1235 (2024). 38870286 10.1126/science.abq6993

[10] Li, G. et al. Structure and performance evolution of perovskite solar cells under extreme temperatures. *Adv. Energy Mater.* **12**, 2202887 (2022).

[11] Li, G. et al. Highly efficient pin perovskite solar cells that endure temperature variations. *Science* **379**, 399–403 (2023). 36701445 10.1126/science.add7331

[12] Duan, T. et al. Chiral-structured heterointerfaces enable durable perovskite solar cells. *Science* **384**, 878–884 (2024). 38781395 10.1126/science.ado5172

[13] Fei, C. et al. Strong-bonding hole-transport layers reduce ultraviolet degradation of perovskite solar cells. *Science* **384**, 1126–1134 (2024). 38843338 10.1126/science.adi4531

[14] Khenkin, M. V. et al. Consensus statement for stability assessment and reporting for perovskite photovoltaics based on ISOS procedures. *Nat. Energy* **5**, 35–49 (2020).

[15] Nie, W. et al. Light-activated photocurrent degradation and self-healing in perovskite solar cells. *Nat. Commun.* **7**, 11574 (2016). 27181192 10.1038/ncomms11574 PMC4873646

[16] Doherty, T. A. et al. Stabilized tilted-octahedra halide perovskites inhibit local formation of performance-limiting phases. *Science* **374**, 1598–1605 (2021). 34941391 10.1126/science.abl4890

[17] Liu, X. et al. Stabilization of photoactive phases for perovskite photovoltaics. *Nat. Rev. Chem.* **7**, 462–479 (2023). 37414982 10.1038/s41570-023-00492-z

[18] Liang, J. et al. Origins and influences of metallic lead in perovskite solar cells. *Joule* **6**, 816–833 (2022).

[19] Wang, L. et al. A Eu^3+^-Eu^2+^ ion redox shuttle imparts operational durability to Pb-I perovskite solar cells. *Science* **363**, 265–270 (2019). 30655439 10.1126/science.aau5701

[20] Rothmann, M. U. et al. Atomic-scale microstructure of metal halide perovskite. *Science* **370**, eabb5940 (2020). 33122356 10.1126/science.abb5940

[21] Gemen, J. et al. Disequilibrating azobenzenes by visible-light sensitization under confinement. *Science* **381**, 1357–1363 (2023). 37733864 10.1126/science.adh9059

[22] Xiong, Q. et al. Managed spatial strain uniformity for efficient perovskite photovoltaics enables minimized energy deficit. *Joule* **8**, 817–834 (2024).

[23] Wang, Q. et al. Tailored succinic acid-derived molecular structures toward 25.41%-efficiency and stable perovskite solar cells. *Adv. Mater.* **36**, 2307709 (2024). 10.1002/adma.202307709 38011852

[24] Wang, R. et al. Constructive molecular configurations for surface-defect passivation of perovskite photovoltaics. *Science* **366**, 1509–1513 (2019). 31857483 10.1126/science.aay9698

[25] Li, Y. et al. Acetic acid assisted crystallization strategy for high efficiency and long-term stable perovskite solar cell. *Adv. Sci.* **7**, 1903368 (2020). 10.1002/advs.201903368 PMC7055551 32154088

[26] Li, F. et al. Hydrogen-bond-bridged intermediate for perovskite solar cells with enhanced efficiency and stability. *Nat. Photonics* **17**, 478–484 (2023).

[27] Mariotti, S. et al. Interface engineering for high-performance, triple-halide perovskite–silicon tandem solar cells. *Science* **381**, 63–69 (2023). 37410849 10.1126/science.adf5872

[28] Wu, L. et al. Stabilization of inorganic perovskite solar cells with a 2D Dion–Jacobson passivating layer. *Adv. Mater.* **35**, 2304150 (2023). 10.1002/adma.202304150 37463023

[29] Yu, S. et al. Homogenized NiO_*x*_ nanoparticles for improved hole transport in inverted perovskite solar cells. *Science* **382**, 1399–1404 (2023). 37995210 10.1126/science.adj8858

[30] Ma, Y. et al. Enhanced interfacial modification by ordered discotic liquid crystals for thermotolerance perovskite solar cells. *Angew. Chem. Int. Ed.* **63**, e202411121 (2024). 10.1002/anie.202411121 39218793

[31] Zhang, Z. et al. Improved air stability of tin halide perovskite solar cells by an N-type active moisture barrier. *Adv. Funct. Mater.* **34**, 2306458 (2024).

[32] Zhang, Z. et al. Anchoring charge selective self-assembled monolayers for tin–lead perovskite solar cells. *Adv. Mater.* **36**, 2312264 (2024). 10.1002/adma.202312264 38281081

[33] Perdew, J. P., Burke, K. & Ernzerhof, M. Generalized gradient approximation made simple. *Phys. Rev. Lett.* **77**, 3865 (1996). 10062328 10.1103/PhysRevLett.77.3865

[34] VandeVondele, J. et al. Quickstep: fast and accurate density functional calculations using a mixed Gaussian and plane waves approach. *Comput. Phys. Commun.* **167**, 103–128 (2005).

[35] Hutter, M. J. et al. cp2k: atomistic simulations of condensed matter systems. *WIREs Comput. Mol. Sci.* **4**, 15–25 (2014).

[36] VandeVondele, J. & Hutter, J. Gaussian basis sets for accurate calculations on molecular systems in gas and condensed phases. *J. Chem. Phys.* **127**, 114105 (2007). 17887826 10.1063/1.2770708

[37] Goedecker, S., Teter, M. & Hutter, J. Separable dual-space Gaussian pseudopotentials. *Phys. Rev. B* **54**, 1703–1710 (1996). 10.1103/physrevb.54.1703 9986014

[38] Carignano, M. A., Kachmar, A. & Hutter, J. Thermal effects on CH_3_NH_3_PbI_3_ perovskite from ab initio molecular dynamics simulations. *J. Phys. Chem. C* **119**, 8991–8997 (2015).

[39] Grimme, S. Semiempirical GGA-type density functional constructed with a long-range dispersion correction. *J. Comput. Chem.* **27**, 1787–1799 (2006). 16955487 10.1002/jcc.20495

[40] Martyna, G. J., Klein, M. L. & Tuckerman, M. Nosé–Hoover chains: the canonical ensemble via continuous dynamics. *J. Chem. Phys.* **97**, 2635–2643 (1992).

[41] Frisch, M. et al. Gaussian 16, Revision C. 01 (Gaussian Inc., 2016).

[42] Chen, H. et al. Improved charge extraction in inverted perovskite solar cells with dual-site-binding ligands. *Science* **384**, 189–193 (2024). 38603485 10.1126/science.adm9474

[43] Wang, H. et al. Interfacial residual stress relaxation in perovskite solar cells with improved stability. *Adv. Mater.* **31**, 1904408 (2019). 10.1002/adma.201904408 31617644

[44] Zhu, S. et al. Multiple dynamic hydrogen bonding networks boost the mechanical stability of flexible perovskite solar cells. *Adv. Funct. Mater.* **34**, 2408487 (2024).

[45] Rolston, N. et al. Engineering stress in perovskite solar cells to improve stability. *Adv. Energy Mater.* **8**, 1802139 (2018).

[46] Reyes-Martinez, M. A. et al. Time-dependent mechanical response of APbX_3_ (A = Cs, CH_3_NH_3_; X = I, Br) single crystals. *Adv. Mater.* **29**, 1606556 (2017). 10.1002/adma.201606556 28464367

[47] Yu, J., Wang, M. & Lin, S. Probing the soft and nanoductile mechanical nature of single and polycrystalline organic-inorganic hybrid perovskites for flexible functional devices. *ACS Nano* **10**, 11044–11057 (2016). 27935297 10.1021/acsnano.6b05913

