# Supplementary material for: Photoswitchable isomers to improve grain boundary resilience and perovskite solar cells stability under light cycling
Source: Nat Energy. 2026 Feb 25;11(4):623–32. doi: 10.1038/s41560-026-01993-z (PMC13121018; doi:10.1038/s41560-026-01993-z)
Supplement: Supplementary file 1 — Supplementary Notes 1–3, Figs. 1–52, Tables 1 and 2 and ref. 1. [file 41560_2026_1993_MOESM1_ESM.pdf]

nature energy

Supplementary information https://doi.org/10.1038/s41560-026-01993-z

Photoswitchable isomers to improve grain boundary resilience and perovskite solar cells stability under light cycling

In the format provided by the authors and unedited

Table of Contents

Supplementary Notes 1-3

Supplementary Figures 1-52

Supplementary Tables 1-2

Supplementary Reference 1

Supplementary Notes

Supplementary Note 1: Device performance under rapid light cycling with varying cycle durations

Rapid light cycling was conducted to determine the behavior of the devices using various cycle times. The devices remained at 73, 77, 83, and 87% of initial PCE after 1200 cycles under xenon lamp light for 30 s light and 30 s dark, 60 s light and 60 s dark, 90 s light and 90 s dark, and 120 s light and 120 s dark, respectively.

Supplementary Note 2: Control experiment to assess SAM stability under UV irradiation

To exclude the potential influence of self-assembled monolayers (SAMs) on device performance during the experimental process, a dedicated control experiment was conducted. Two representative SAM molecules—[4-(3,6-dimethoxy-9H-carbazol-9-yl)butyl]phosphonic acid (MeO-4PACz) and 4-(9H-carbazol-9-yl)phenylphosphonic acid (4PACz)—were first subjected to ultraviolet (UV) light irradiation (365 nm) for approximately 240 hours under identical environmental conditions to those used in the device stability tests. After UV pre-aging, perovskite solar cells were fabricated using these aged SAMs as the hole-transporting interfacial layer. In parallel, devices based on freshly prepared (unaged) SAMs were fabricated under the same conditions and used as control samples.

The photovoltaic parameters of the resulting devices were measured before and after UV exposure and compared. As shown in Supplementary Fig. 4, the devices fabricated with UV-aged SAMs exhibited only a slight reduction in power conversion efficiency, open-circuit voltage, and fill factor relative to the control devices. In contrast, devices fabricated with unaged SAMs and subsequently subjected to UV light aging displayed a significantly more pronounced degradation in all photovoltaic metrics.

These comparative results clearly demonstrate that the observed device performance decay primarily originates from the degradation of the perovskite absorber layer rather than from the deterioration of the SAMs themselves. Therefore, the influence of SAM degradation on device performance during the experimental process can be reasonably excluded.

Supplementary Note 3: Transition-state and IRC analysis of *E*–*Z* isomerization

To clarify this process, we employed TS calculations to determine the single-molecule activation barrier, and performed intrinsic reaction coordinate (IRC) analyses to trace the paths from the TS to both *E* and *Z* configurations. High-level CCSD(T) calculations were used to obtain the reaction energy profile (Supplementary Fig. 11). The results indicate that the *E*→*Z* conversion is feasible under light illumination. In the TS structure, the N=N–C bond angle is close to 180°, consistent with literature reports[1], confirming that the isomerization occurs predominantly via rotation around the N=N bond.

Supplementary figures

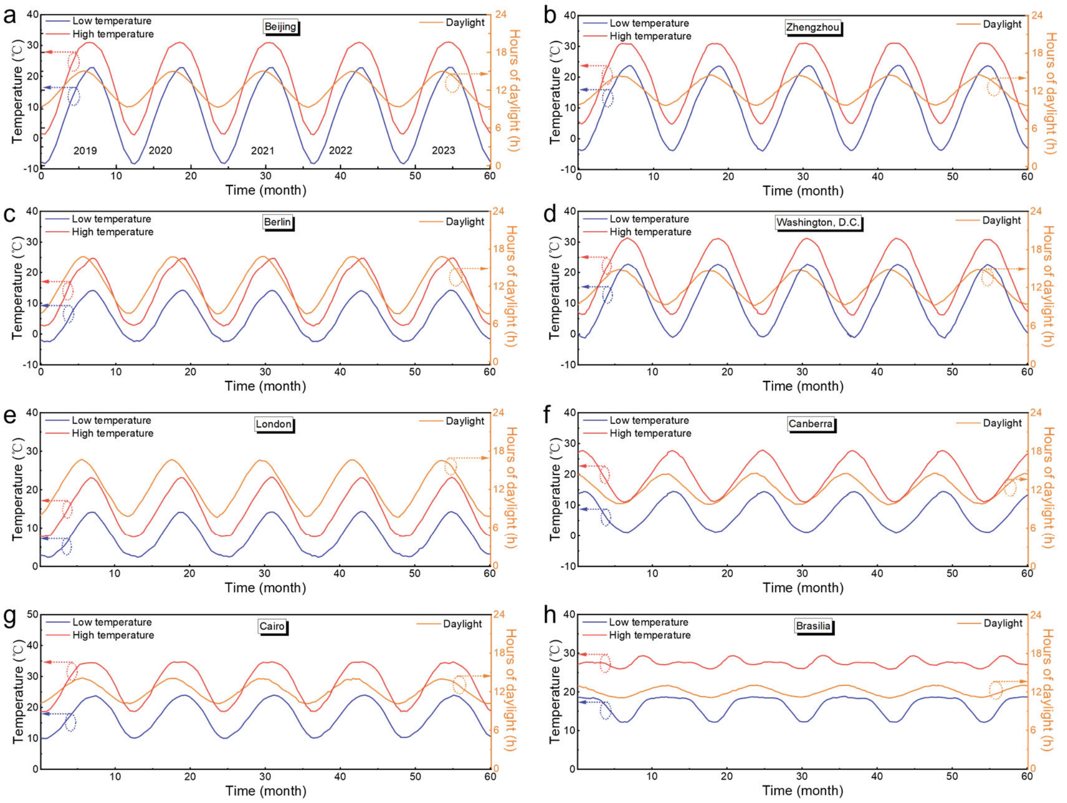

Supplementary Fig. 1. Temperature and hours of sunlight for (a) Beijing (Asia), (b) Zhengzhou (our laboratory, Asia), (c) Berlin (HZB, Europe), (d) Washington, D. C. (North America), (e) London (Europe), (f) Canberra (Oceania), (g) Cairo (Africa) and (h) Brasilia (South America) over the last five years.

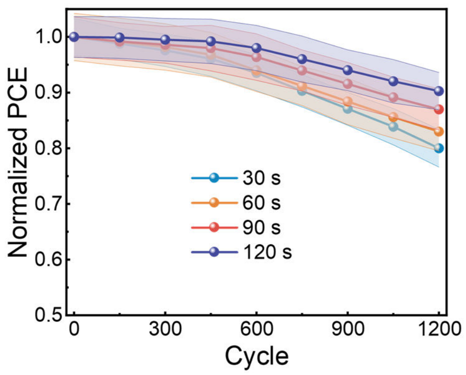

Supplementary Fig. 2. Light cycling with a xenon lamp light for 30 s light and 30 s dark, 60 s light and 60 s dark, 90 s light and 90 s dark, and 120 s light and 120 s dark. Solid lines show mean values, and shaded areas indicate mean ± SD. The initial average PCEs for different light cycling groups were 24.8% ± 0.83% (30 s), 25.0% ± 1.04% (60 s), 24.9% ± 0.90% (90 s), and 24.5% ± 0.90% (120 s), respectively (n = 6).

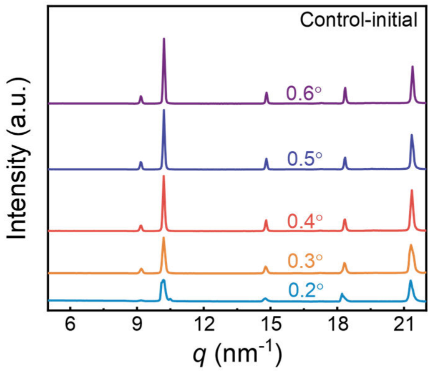

Supplementary Fig. 3. GIWAXS 1D integrated curves of the control-initial perovskite film at different incident angles.

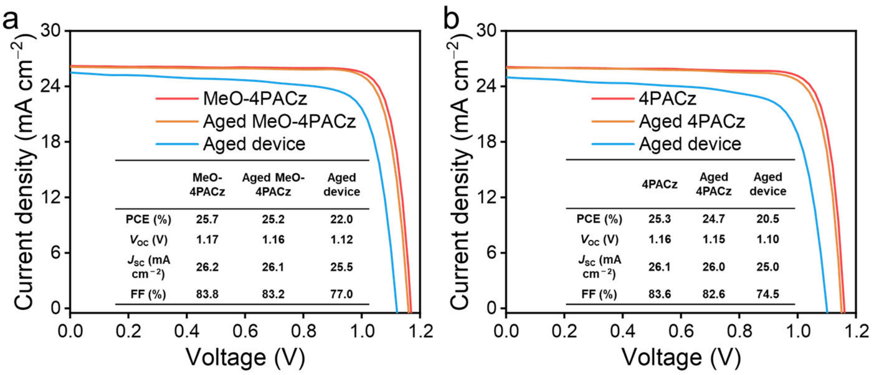

Supplementary Fig. 4. *J-V* curves of (a) MeO-4PACz relative devices, and (b) 4PACz relative devices.

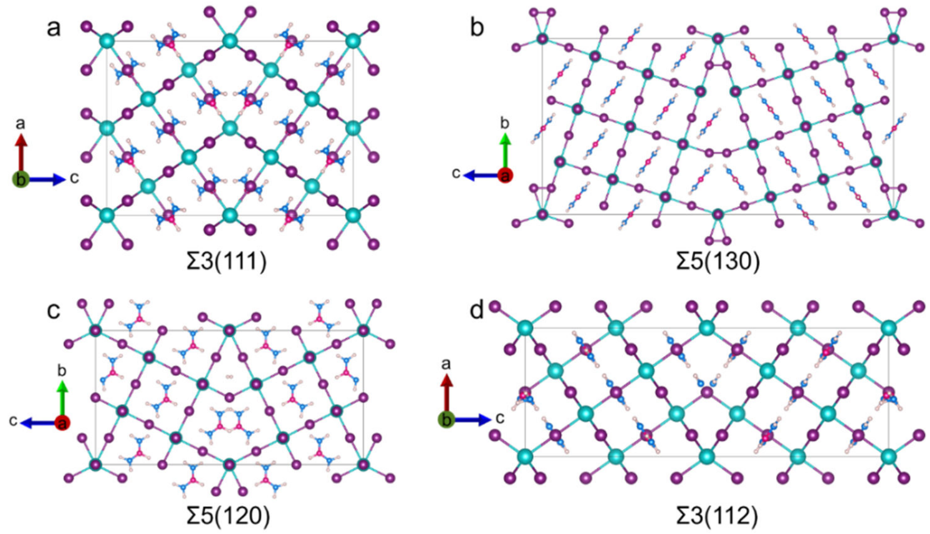

Supplementary Fig. 5. Four grain boundary structures constructed based on the CSL theory: (a) Σ3(111), (b) Σ5(130), (c) Σ5(120), (d) Σ3(112). Pb and I atoms are shown as cyan and purple spheres, respectively; the organic ions are shown in a ball-and-stick representation (C, pink/magenta; N, blue; H, white).

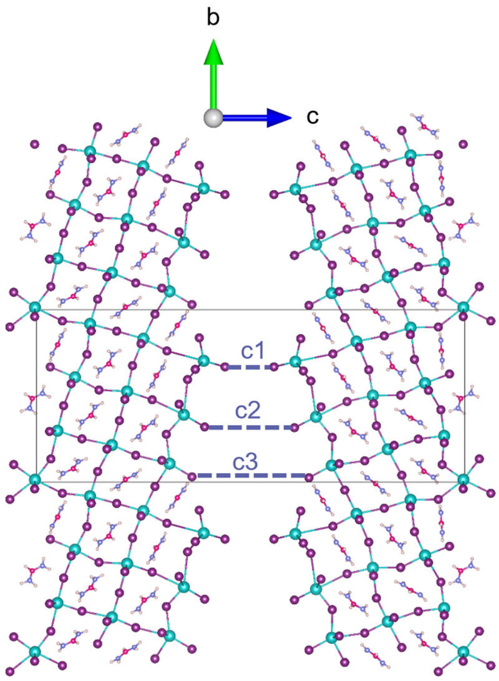

Supplementary Fig. 6. Schematic diagram of a grain boundary, where c1, c2, and c3 represent a crystal point. Pb and I atoms are shown as cyan and purple spheres, respectively; the organic ions are shown in a ball-and-stick representation (C, pink/magenta; N, blue; H, white). The dashed lines (c1–c3) mark three representative lattice sites at the GB where the interfacial spacing is evaluated.

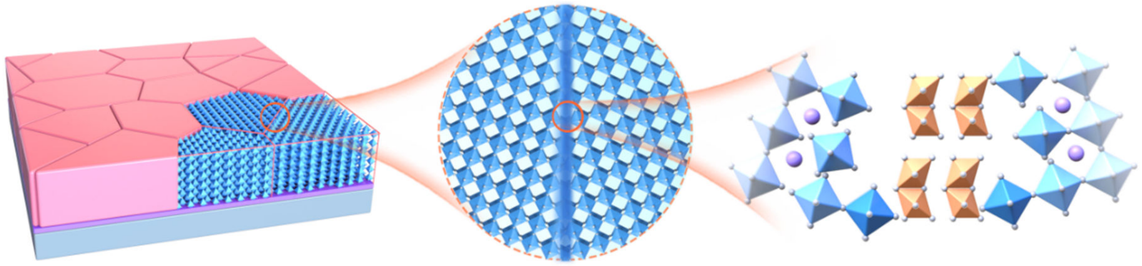

Supplementary Fig. 7. Representation of the perovskite film, crystal lattice, and grain boundary. Blue polyhedra denote the perovskite $[PbI_6]^{4-}$ octahedra, purple spheres denote A^+ site cations (FA^+/MA^+/Cs^+), and orange polyhedra denote PbI_2.

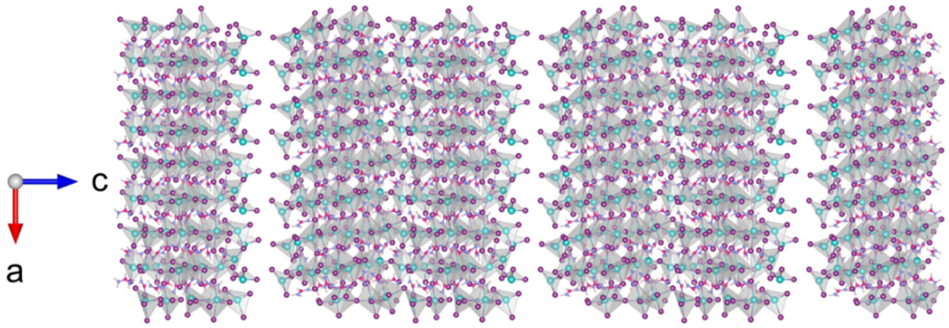

Supplementary Fig. 8. Calculated the perovskite grain boundary under a simulated energy field (side view). Pb and I atoms are shown as cyan and purple spheres, respectively; the organic ions are shown in a ball-and-stick representation (C, pink/magenta; N, blue; H, white).

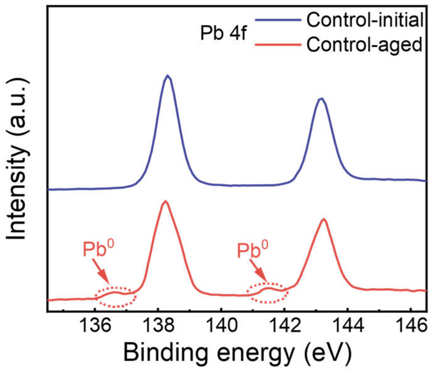

Supplementary Fig. 9. Pb 4f XPS spectra of the control perovskite film before and after 80 light cycles (12 hours in light and 12 hours in dark, xenon lamp light).

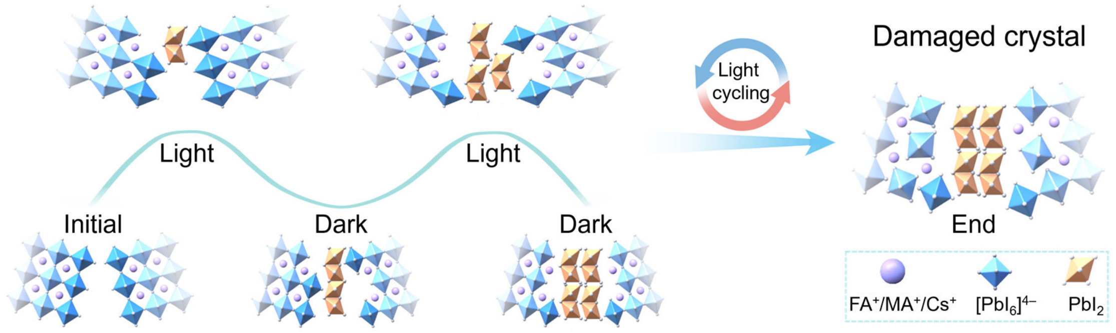

Supplementary Fig. 10. Schematic of perovskite decay under light cycling.

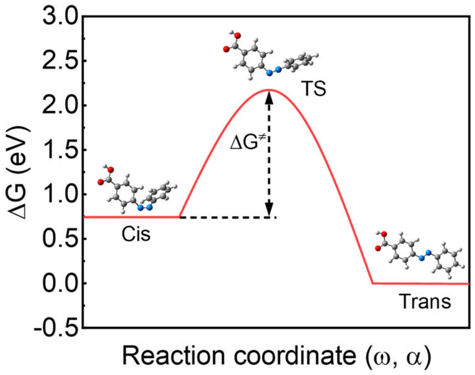

Supplementary Fig. 11. Free energy profile for *E*-to-*Z* conformational isomerization. Vertical axis: ΔG (eV); horizontal axis: reaction coordinate (defined by dihedrals ω and α). The curve shows the energy barrier ($\Delta G^{\neq}$) via the TS from the cis to the more stable trans isomer.

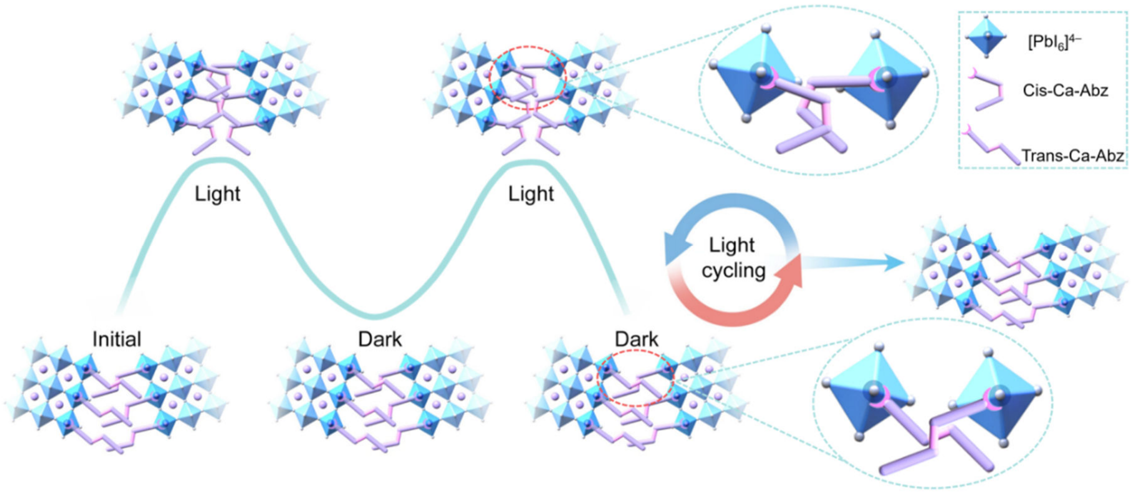

Supplementary Fig. 12. Schematic showing the role of Ca-Abz as a buffer in the perovskite grain boundary under light cycling.

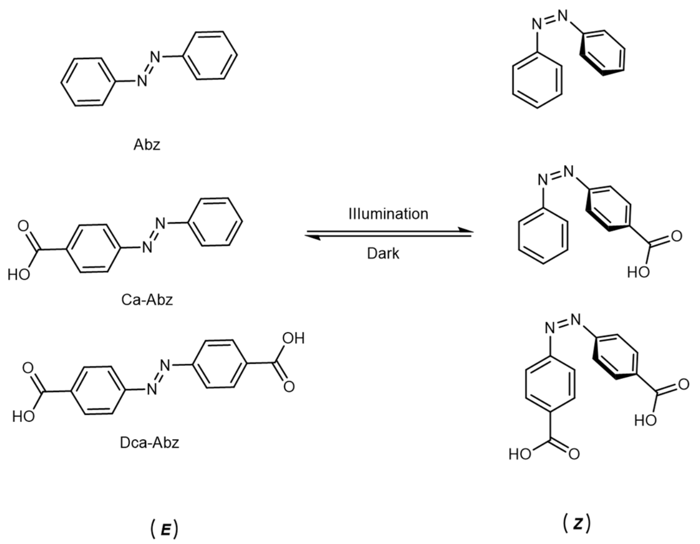

Supplementary Fig. 13. Generic illustration and the structures of the *E* and *Z* isomers of azobenzene (Abz), 4-(phenylazo)benzoic acid (Ca-Abz), and azobenzene-4,4'-dicarboxylic acid (Dca-Abz) under xenon lamp light and dark conditions.

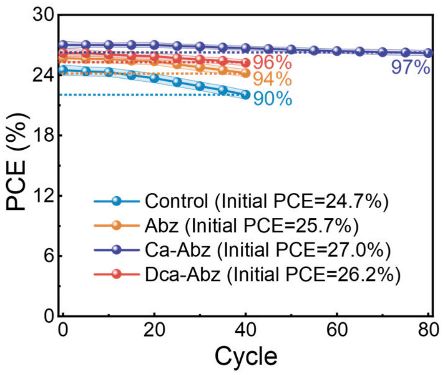

Supplementary Fig. 14. The light cycling stability of control, Abz-, Ca-Abz-, and Dca-Abz-based devices (12 hours light and 12 hours dark). Solid lines show mean values, and shaded areas indicate mean ± SD. The initial average PCEs for the different device groups were 24.5% ± 0.52% (control), 25.7% ± 0.36% (Abz), 27.0% ± 0.35% (Ca-Abz), and 26.2% ± 0.33% (Dca-Abz), respectively (n = 6).

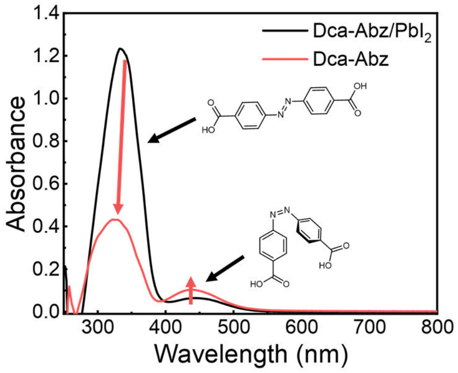

Supplementary Fig. 15. UV-vis spectra of Dca-Abz/PbI_2 and Dca-Abz.

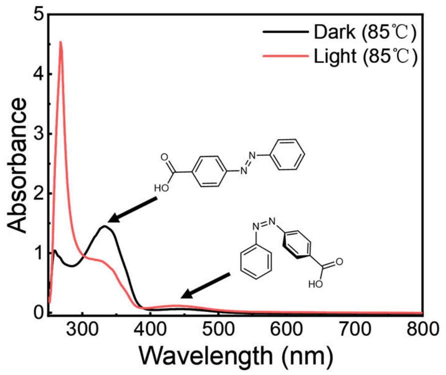

Supplementary Fig. 16. UV-vis spectra of Ca-Abz in dark (85°C) and light (85°C).

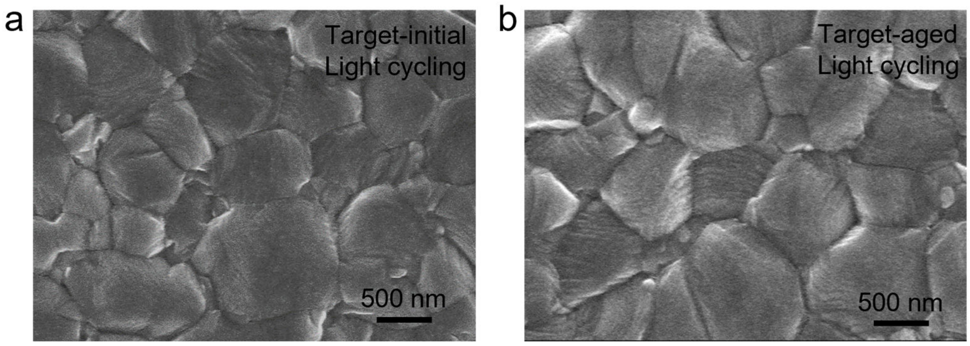

Supplementary Fig. 17. SEM images of the target perovskite film (a) before and (b) after 80 light cycles (12 hours in light and 12 hours in dark, xenon lamp light).

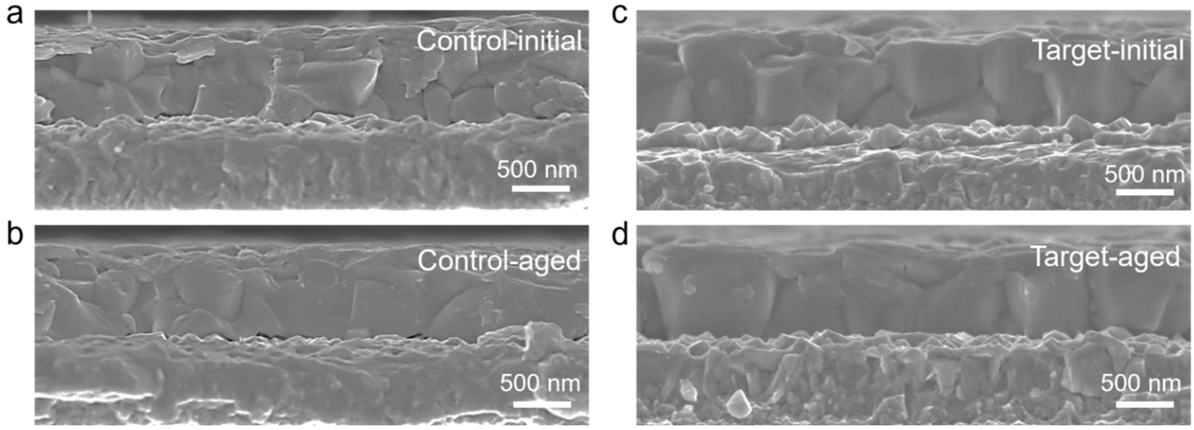

Supplementary Fig. 18. Cross-section SEM images of the (a) control-initial, (b) control-aged, (c) target-initial, and (d) target-aged perovskite films. The perovskite films were ageing at light cycling for 80 cycles (12 hours in light and 12 hours in dark, xenon lamp light). From the cross-section SEM images, the cracks in the control film and the undamaged morphology in the target film indicate the effectiveness of the strain-buffer coupling strategy using Ca-Abz.

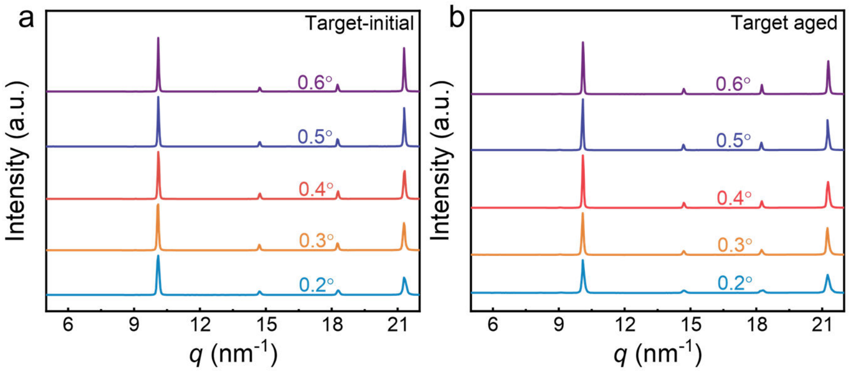

Supplementary Fig. 19. GIWAXS 1D integrated curves of the (a) initial and (b) aged target perovskite films at different incident angles. The perovskite films were ageing at light cycling (12 hours in light and 12 hours in dark, xenon lamp light).

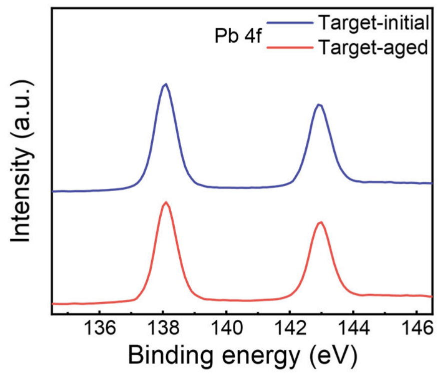

Supplementary Fig. 20. Pb 4f XPS spectra of the target perovskite film before and after 80 light cycles. (12 hours in light and 12 hours in dark, xenon lamp light).

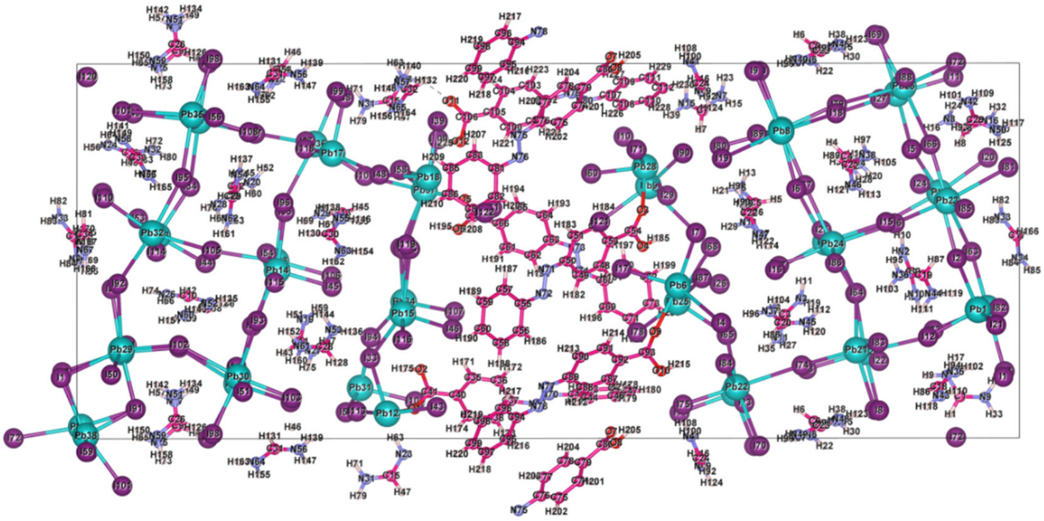

Supplementary Fig. 21. Calculated perovskite grain boundary with Ca-Abz (*E* isomer) under a simulated energy field (top view). Lattice parameters include a=12.826 Å, b=19.665 Å, c=49.523 Å, α=90.072°, β=88.856°, γ=90.527°. Pb and I atoms are shown as cyan and purple spheres, respectively; the organic ions are shown in a ball-and-stick representation (C, pink/magenta; N, blue; H, white).

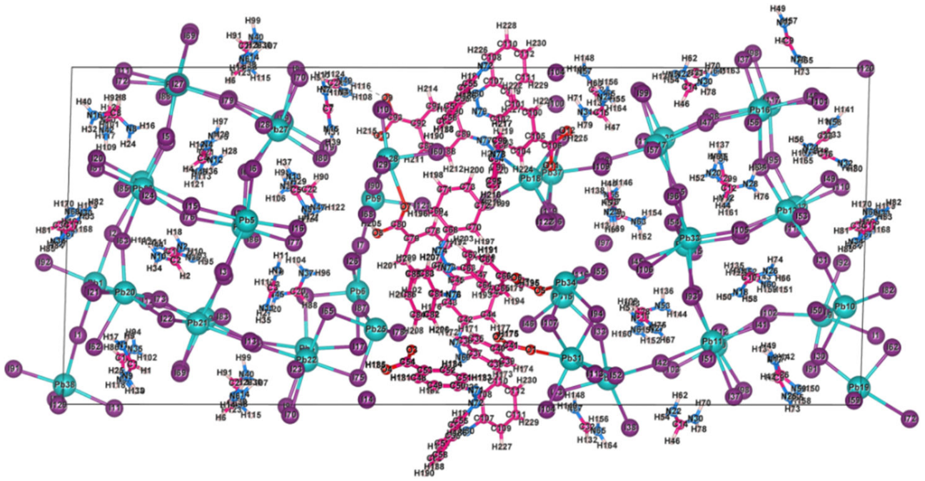

Supplementary Fig. 22. Calculated perovskite grain boundary with Ca-Abz (*Z* isomer) under a simulated energy field (top view). Lattice parameters include a=12.977 Å, b=19.925 Å, c=47.160 Å, α=88.430°, β=90.162°, γ=88.867°. Pb and I atoms are shown as cyan and purple spheres, respectively; the organic ions are shown in a ball-and-stick representation (C, pink/magenta; N, blue; H, white).

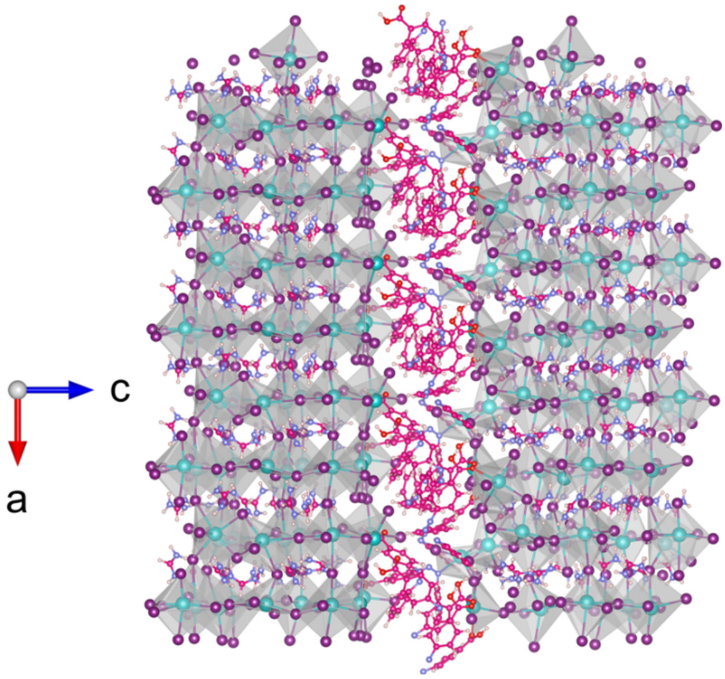

Supplementary Fig. 23. Calculated perovskite grain boundary with Ca-Abz (*Z* isomer) under a simulated energy field (side view). Atom colours: Pb (cyan), I (purple), C (pink), N (blue), O (red), and H (white).

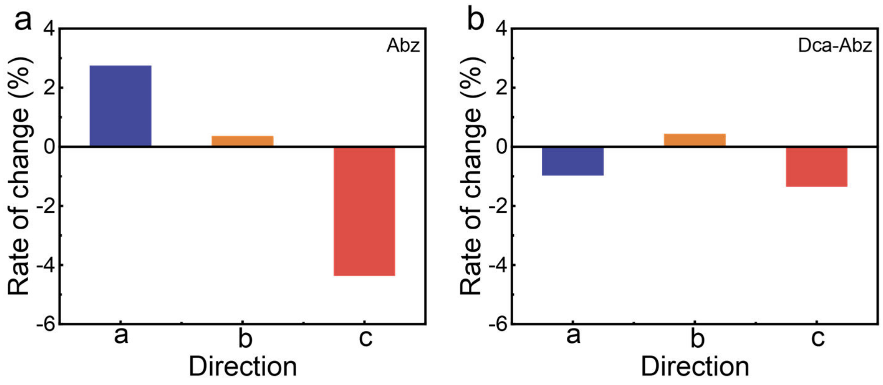

Supplementary Fig. 24. Lattice parameter variation of perovskite modified with (a) Abz and (b) Dca-Abz along the a, b, and c axes.

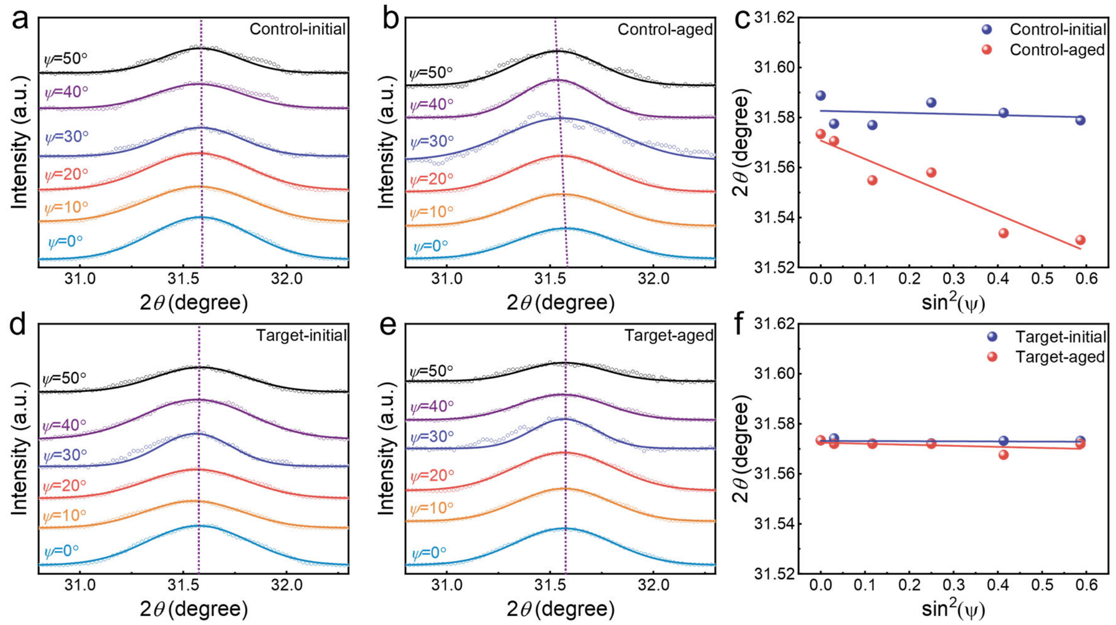

Supplementary Fig. 25. GIXRD spectra of different instrumental ψ values for the control (a) initial and (b) aged films. (c) Linear fit of 2θ as a function of $\sin^2(\psi)$. GIXRD spectra of different instrumental ψ values for the target (d) fresh and (e) aged films. (f) Linear fit of 2θ as a function of $\sin^2(\psi)$.

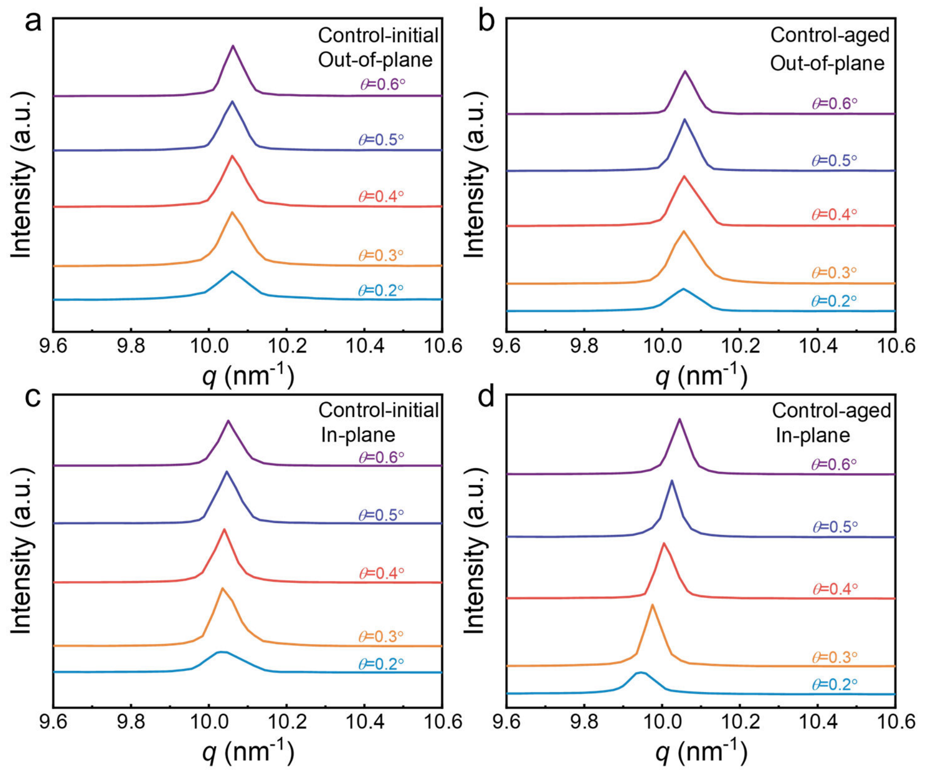

Supplementary Fig. 26. GIWAXS 1D integral curves at different glancing incidence angles for the (a) initial (out-of-plane), (b) aged (out-of-plane), (c) initial (in-plane), and (d) aged (in-plane) control perovskite films.

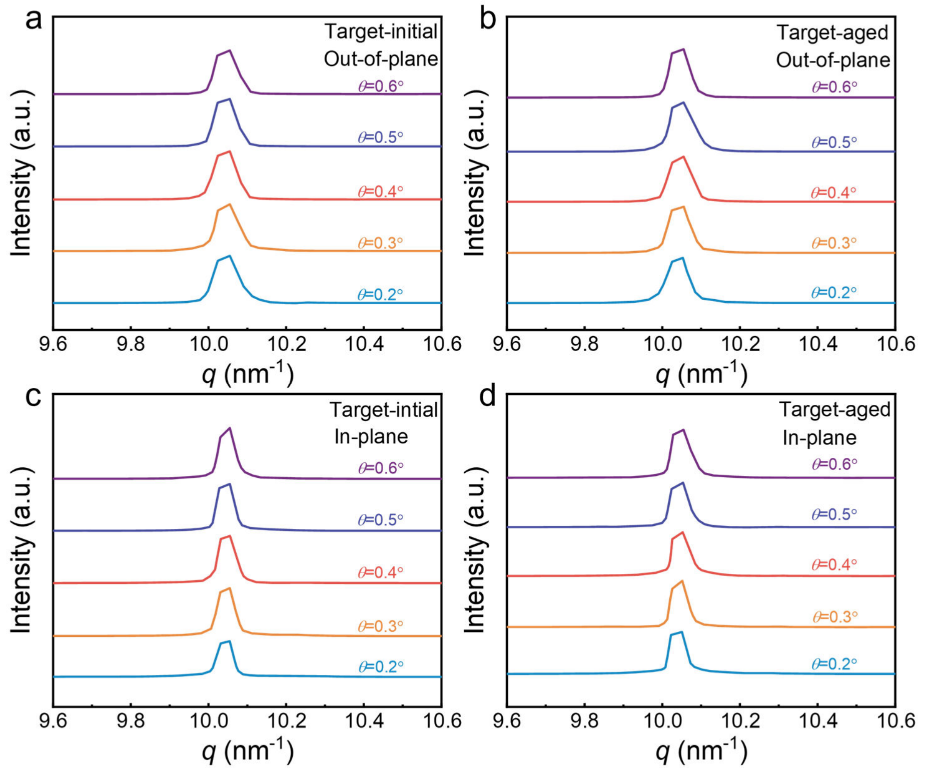

Supplementary Fig. 27. GIWAXS 1D integral curves at different glancing incidence angles for the (a) initial (out-of-plane), (b) aged (out-of-plane), (c) initial (in-plane), and (d) aged (in-plane) target perovskite films.

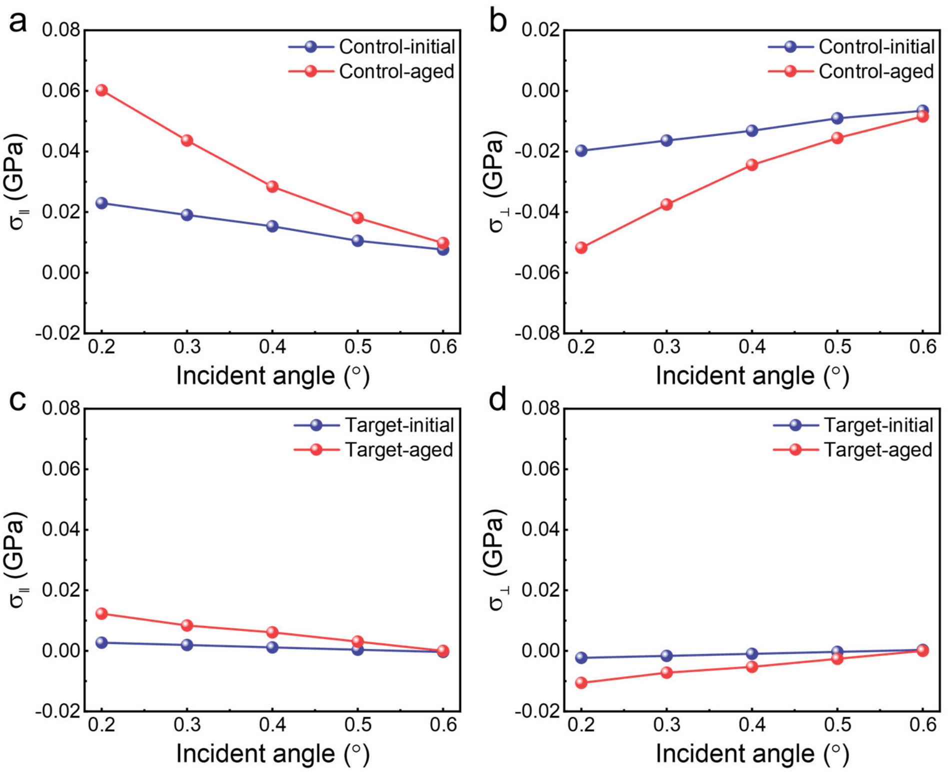

Supplementary Fig. 28. (a) $\sigma_\parallel$ and (b) $\sigma_\perp$ measured at different incident angles for the control films. (c) $\sigma_\parallel$ and (d) $\sigma_\perp$ measured at different incident angles for the target films. The $\sigma_\perp$ is a theoretical value approximated from the in-plane stress $\sigma_\parallel$ based on the assumption of isotropic elasticity.

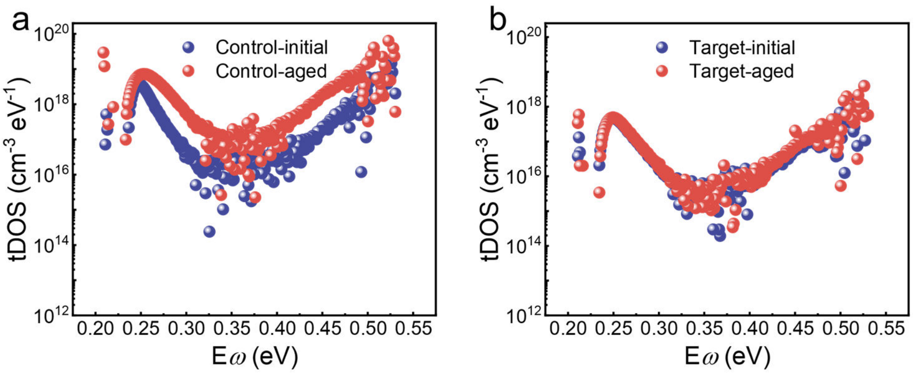

Supplementary Fig. 29. (a) tDOS of control and (b) target devices before and after light cycling for 20 cycles.

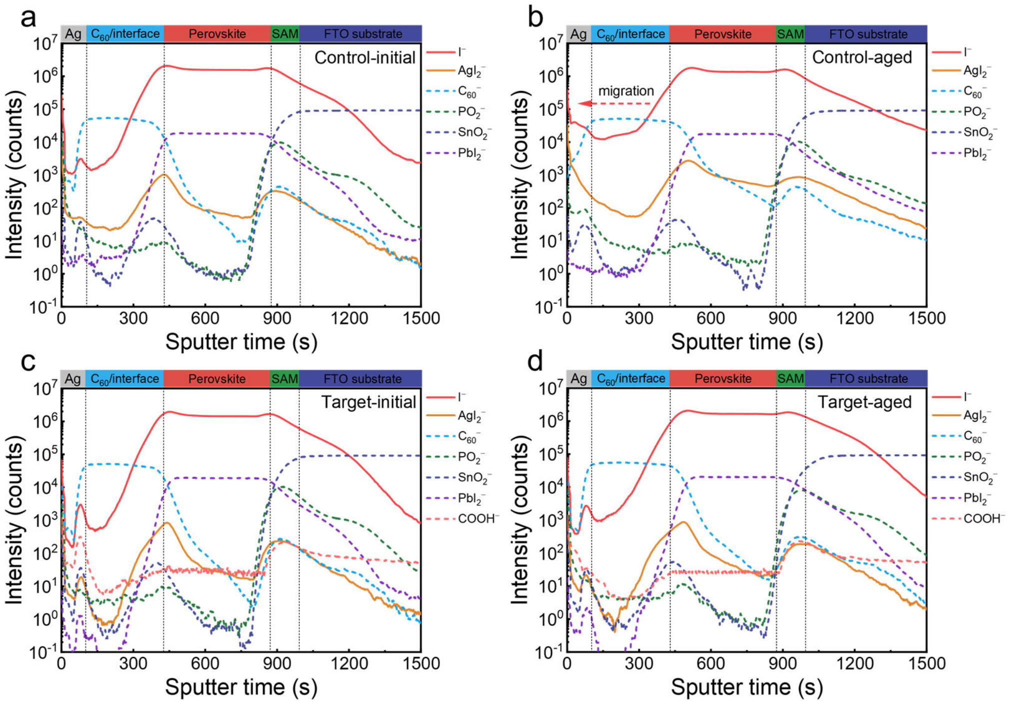

Supplementary Fig. 30. ToF-SIMS profiles of control and target (including Ca-Abz) devices. (a) Initial and (b) aged state of control devices (20 cycles). (c) Initial and (d) aged state of target devices (20 cycles).

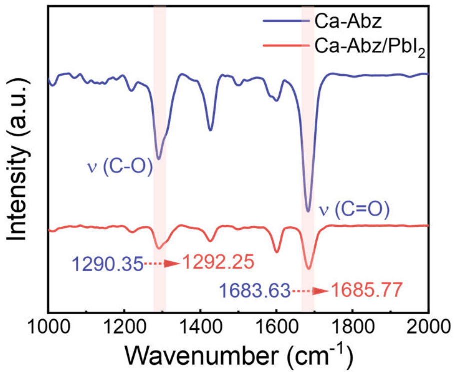

Supplementary Fig. 31. FTIR spectra of Ca-Abz and Ca-Abz/PbI_2 powders.

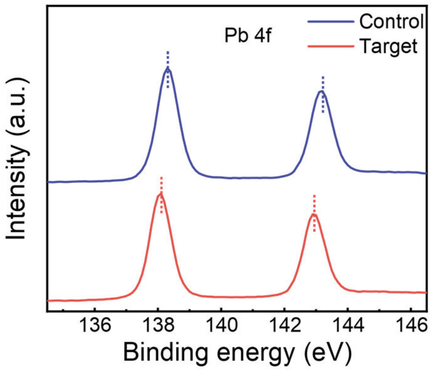

Supplementary Fig. 32. XPS spectra of Pb 4f of the control and target perovskite films.

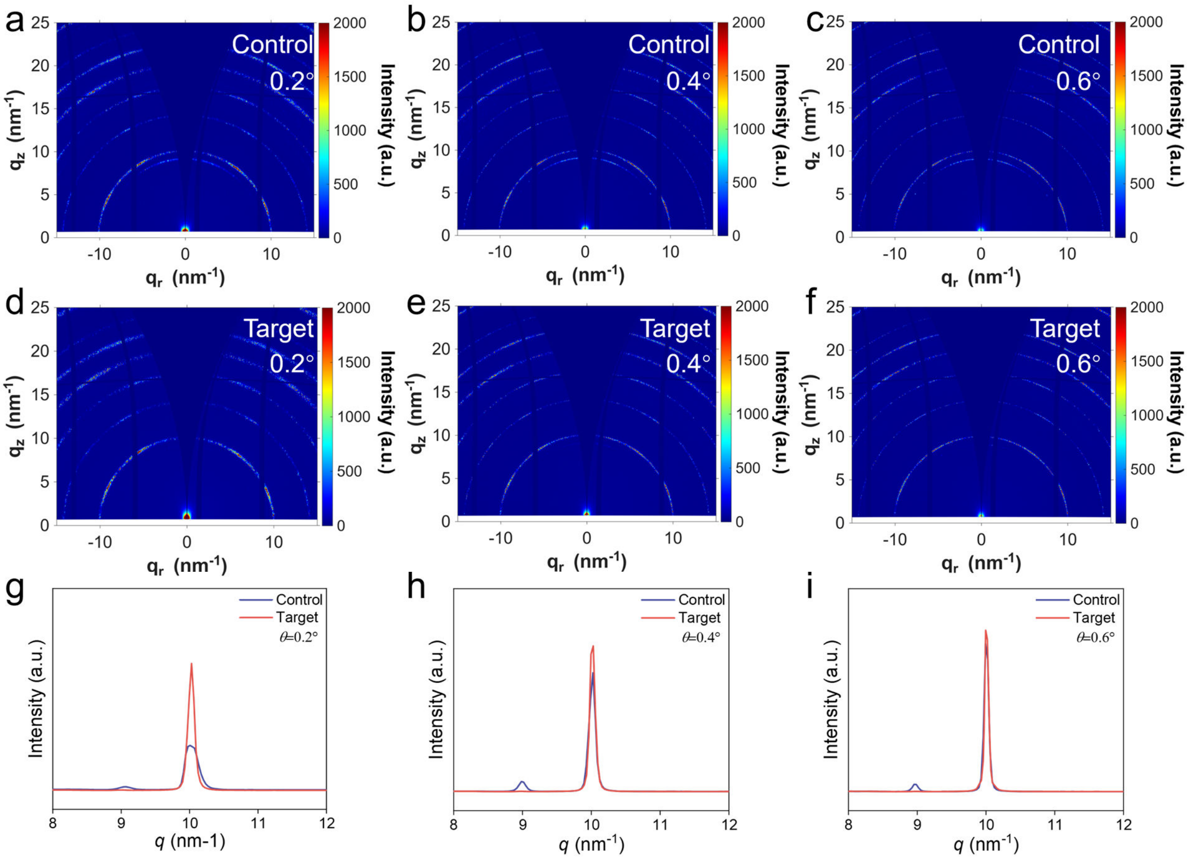

Supplementary Fig. 33. 2D GIWAXS patterns of the control perovskite films at incident angles of (a) 0.2°, (b) 0.4°, and (c) 0.6°. 2D GIWAXS patterns of the target perovskite films at incident angles of (d) 0.2°, (e) 0.4°, and (f) 0.6°. GIWAXS 1D integral curves of an incident angle of (g) 0.2°, (h) 0.4°, and (i) 0.6°.

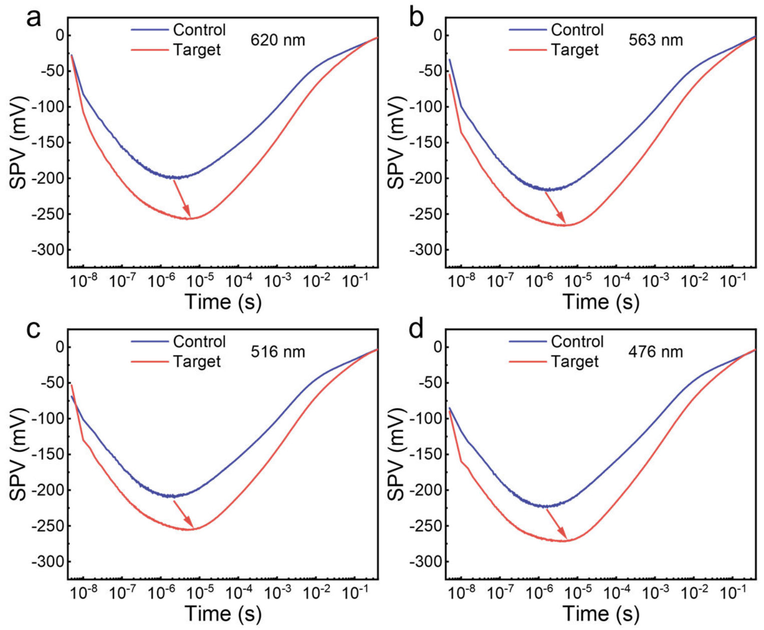

Supplementary Fig. 34. trSPV measurements of devices with the structure substrate/SAM/perovskite/C_{60}/SnOx under (a) 620 nm, (b) 563nm, (c) 516 nm, and (d) 476 nm light laser.

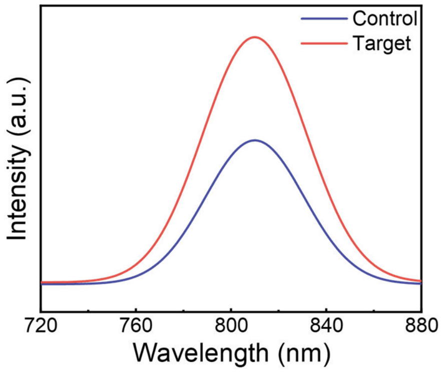

Supplementary Fig. 35. PL spectra of quartz/perovskites (control and target).

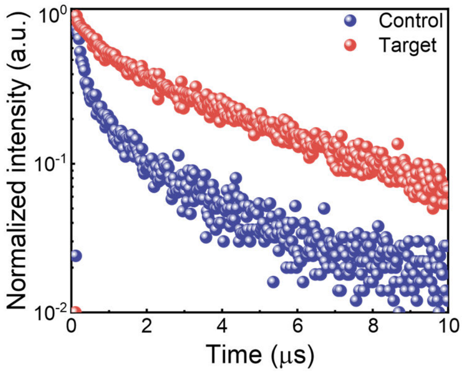

Supplementary Fig. 36. TRPL spectra of quartz/perovskite (control and target), the lifetimes of control and target samples were 966.83 and 3564.04 ns, respectively.

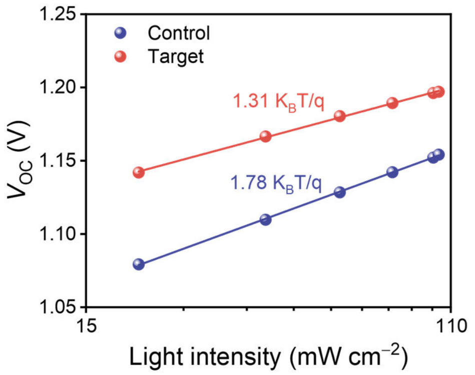

Supplementary Fig. 37. V_{OC} dependence of light intensity for the control and target devices.

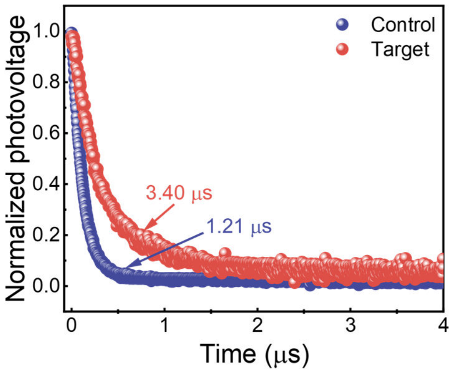

Supplementary Fig. 38. Transient photovoltage of the control and target devices.

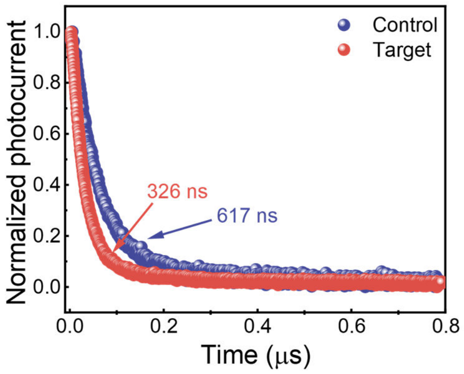

Supplementary Fig. 39. Transient photocurrent of the control and target devices.

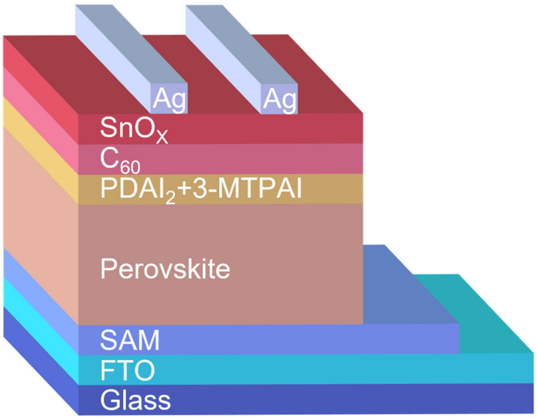

Supplementary Fig. 40. Device architecture used in this work.

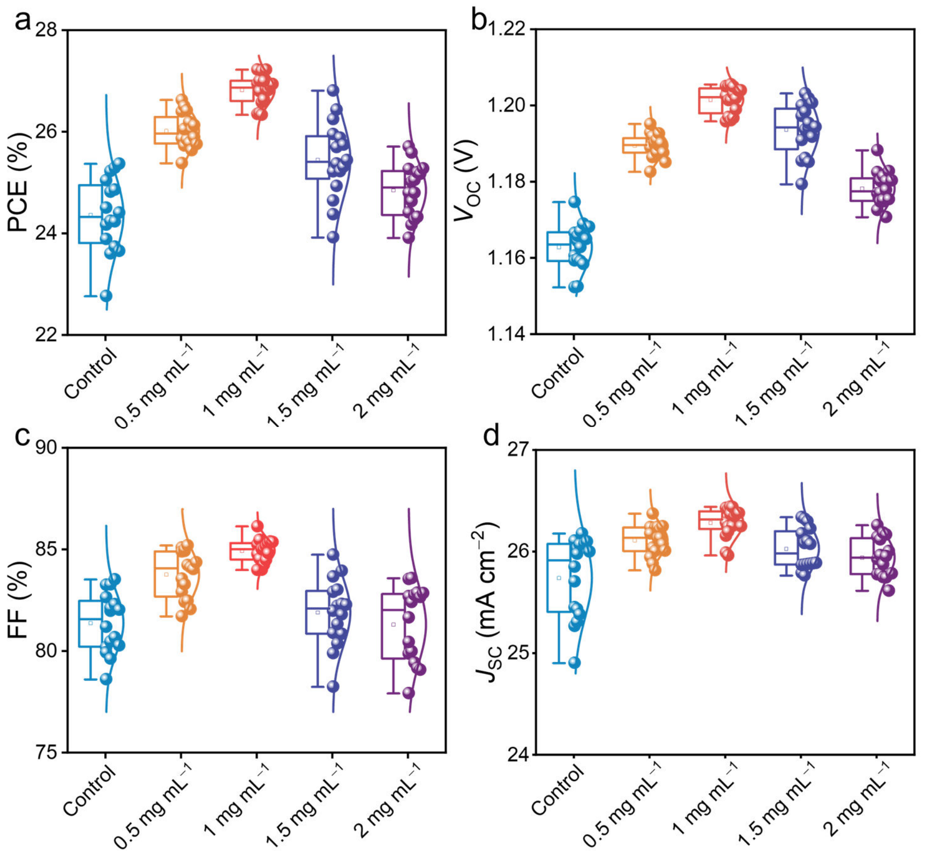

Supplementary Fig. 41. Statistical data for (a) PCE, (b) V_{OC}, (c) FF, and (d) J_{SC}. Box plots show median (centre line), mean (open square), 25th and 75th percentiles (box bounds), and minima/maxima within 1.5× interquartile range (whiskers); dots represent individual devices (n = 16 independent devices per group).

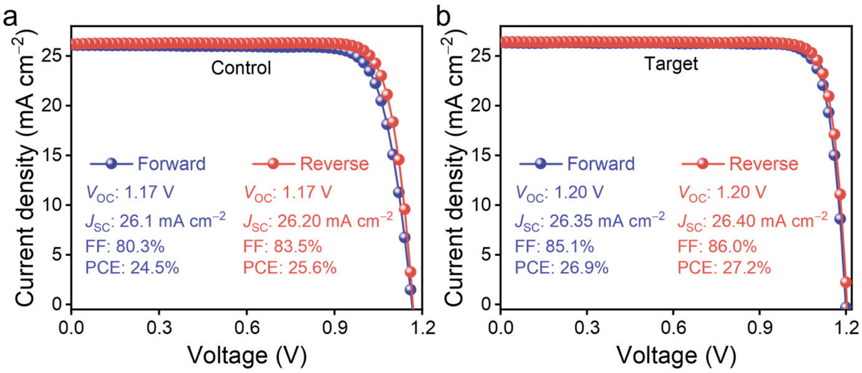

Supplementary Fig. 42. *J*-*V* curves of the (a) control and (b) target devices.

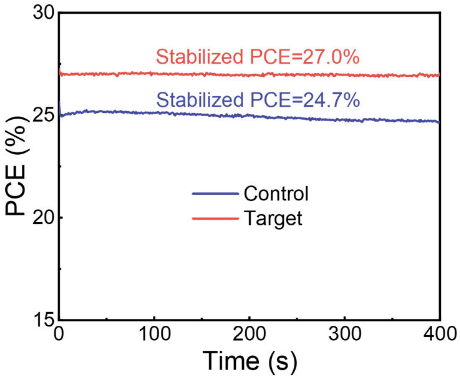

Supplementary Fig. 43. Stabilized power output PCE of the control and target devices.

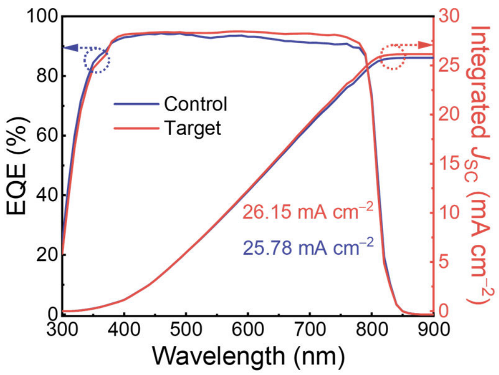

Supplementary Fig. 44. EQE spectra of the control and target devices.

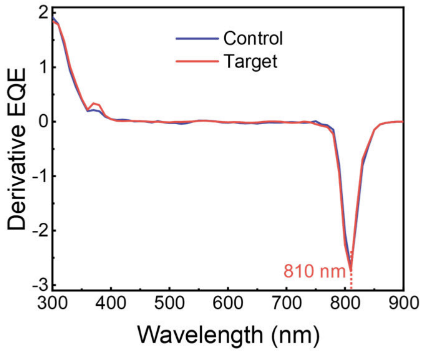

Supplementary Fig. 45. Bandgap of the control and target perovskite films calculated from the derivative of EQE with respect to wavelength.

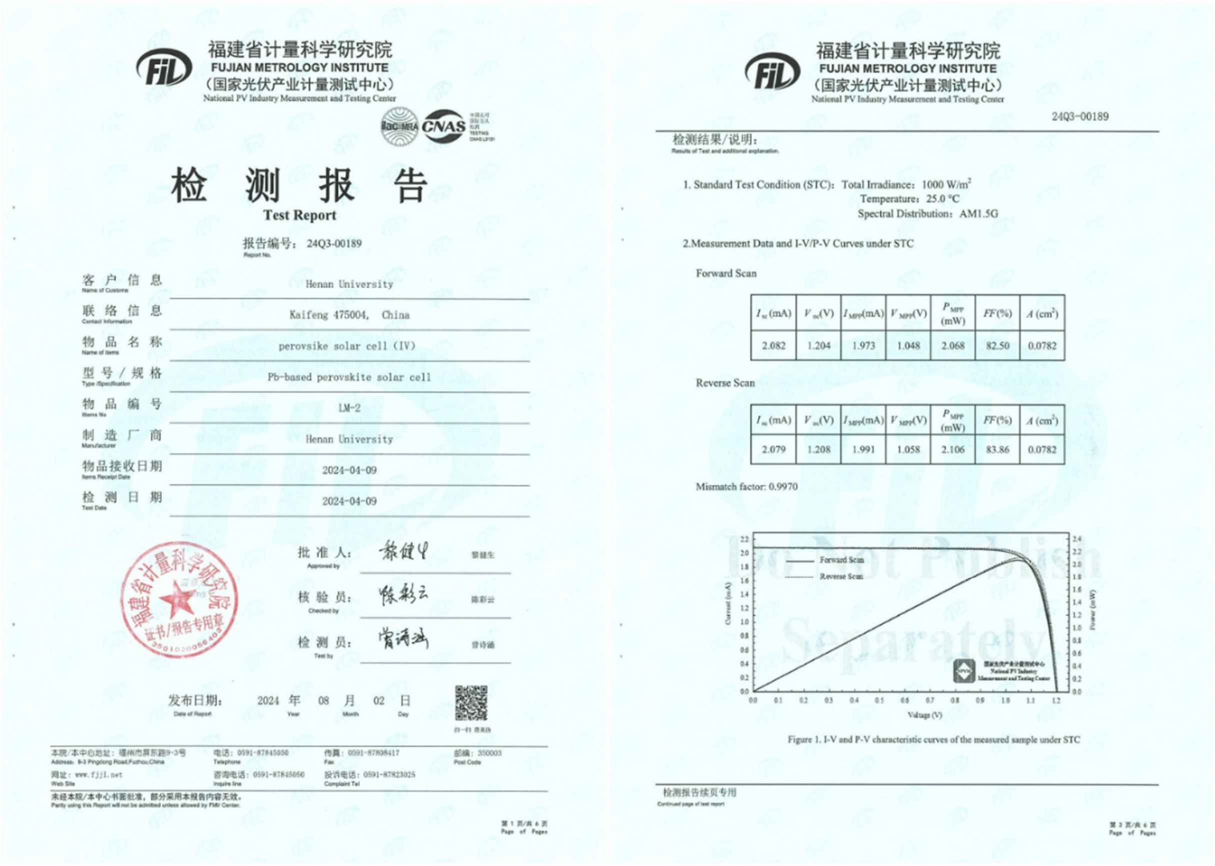

福建省计量科学研究院
FUJIAN METROLOGY INSTITUTE
（国家光伏产业计量测试中心）
National PV Industry Measurement and Testing Center

检 测 报 告
Test Report

报告编号：24Q3-00189
Report No.

客户信息 Name of Customer	Henan University
联络信息 Contact Information	Kaifeng 475004, China
物品名称 Name of Items	perovsike solar cell (IV)
型号/规格 Type /Specification	Pb-based perovskite solar cell
物品编号 Items No	LM-2
制造厂商 Manufacturer	Henan University
物品接收日期 Items Receipt Date	2024-04-09
检测日期 Test Date	2024-04-09

批准人：Approved by 黎健生
核验员：Checked by 陈彩云
检测员：Test by 曾诗涵

发布日期：2024 年 08 月 02 日
Date of Report Year Month Day

本院/本中心地址：福州市屏东路9-3号 Address: 9-3 Pingdong Road,Fuzhou,China
电话：0591-87845050 Telephone
传真：0591-87808417 Fax
邮编：350003 Post Code
网址：www.fjjl.net Web Site
咨询电话：0591-87845050 Inquire line
投诉电话：0591-87823025 Complaint Tel

未经本院/本中心书面批准，部分采用本报告内容无效。
Partly using this Report will not be admitted unless allowed by FMI Center.

第 1 页/共 6 页
Page of Pages

福建省计量科学研究院
FUJIAN METROLOGY INSTITUTE
（国家光伏产业计量测试中心）
National PV Industry Measurement and Testing Center

24Q3-00189

检测结果/说明：
Results of Test and additional explanation.

1. Standard Test Condition (STC): Total Irradiance: 1000 W/m^2
Temperature: 25.0 °C
Spectral Distribution: AM1.5G

2.Measurement Data and I-V/P-V Curves under STC

Forward Scan

I_{sc} (mA)	V_{oc} (V)	I_{MPP} (mA)	V_{MPP} (V)	P_{MPP} (mW)	FF (%)	A (cm^2)
2.082	1.204	1.973	1.048	2.068	82.50	0.0782

Reverse Scan

I_{sc} (mA)	V_{oc} (V)	I_{MPP} (mA)	V_{MPP} (V)	P_{MPP} (mW)	FF (%)	A (cm^2)
2.079	1.208	1.991	1.058	2.106	83.86	0.0782

Mismatch factor: 0.9970

Figure 1. I-V and P-V characteristic curves of the measured sample under STC

检测报告续页专用
Continued page of test report

第 3 页/共 6 页
Page of Pages

Supplementary Fig. 46. The certified *J-V* curves of a Ca-Abz modified (target) device were determined by NPVM (Chinese National PV Industry Measurement and Testing Center).

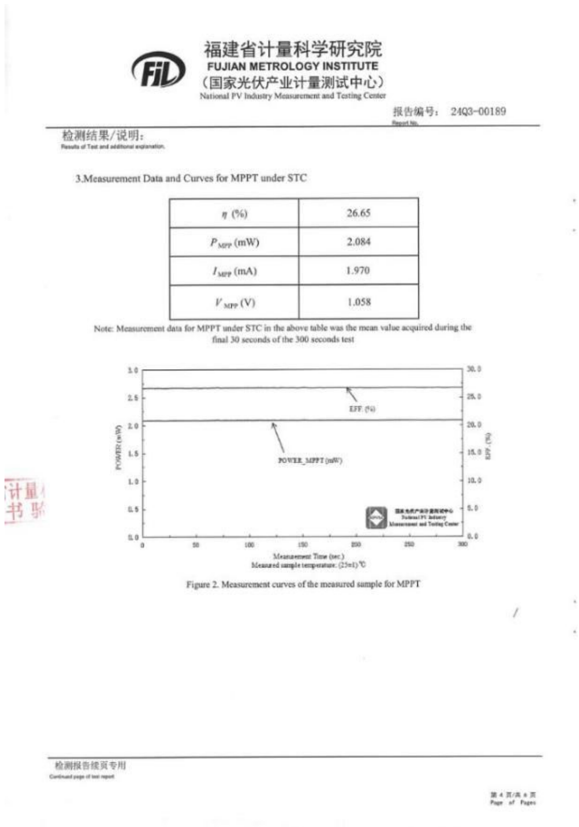

福建省计量科学研究院
FUJIAN METROLOGY INSTITUTE
（国家光伏产业计量测试中心）
National PV Industry Measurement and Testing Center

报告编号： 24Q3-00189
Report No.

检测结果/说明：
Results of Test and additional explanation.

3.Measurement Data and Curves for MPPT under STC

η (%)	26.65
P_{MPP} (mW)	2.084
I_{MPP} (mA)	1.970
V_{MPP} (V)	1.058

Note: Measurement data for MPPT under STC in the above table was the mean value acquired during the final 30 seconds of the 300 seconds test

Figure 2. Measurement curves of the measured sample for MPPT

检测报告续页专用
Continued page of test report

Page of Pages

Supplementary Fig. 47. Certified stabilized PCE of the Ca-Abz modified (target) device by MPPT.

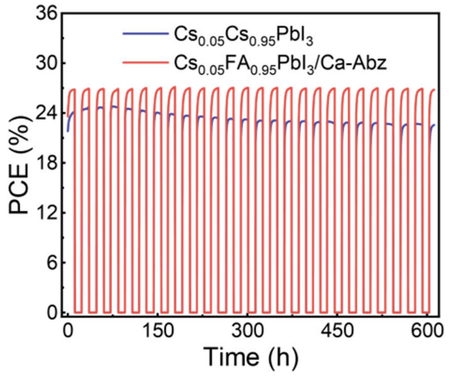

Supplementary Fig. 48. Light cycling of $Cs_{0.05}FA_{0.95}PbI_3$-based devices under the solar simulator (12 hours of light and 12 hours of dark).

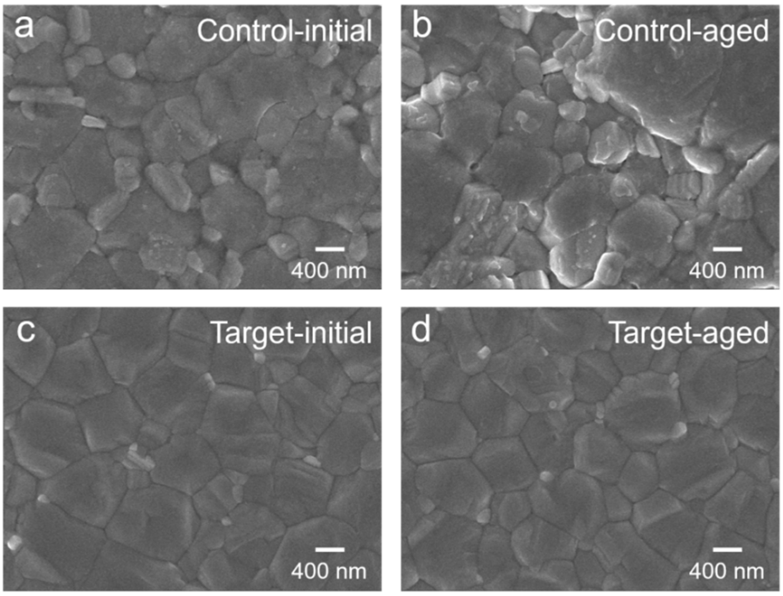

Supplementary Fig. 49. Top-view SEM images of perovskite films. (a) Initial, and (b) aged state of control perovskite films (20 cycles). (c) Initial, and (d) aged state of target perovskite films (20 cycles).

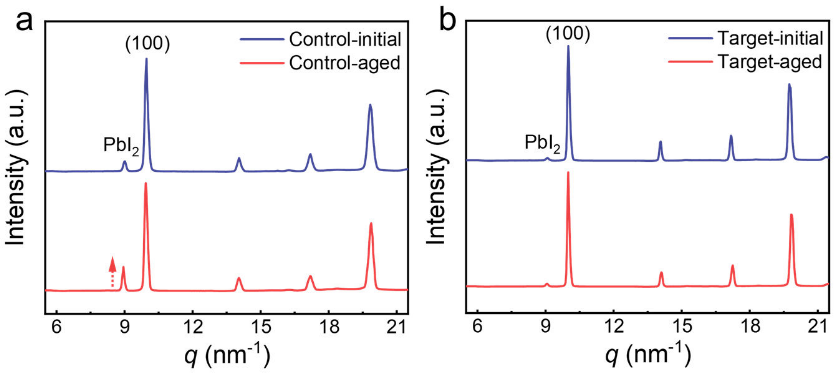

Supplementary Fig. 50. GIWAXS 1D integrated curves of initial and after 20 light cycles aged (a) control, (b) target perovskite films.

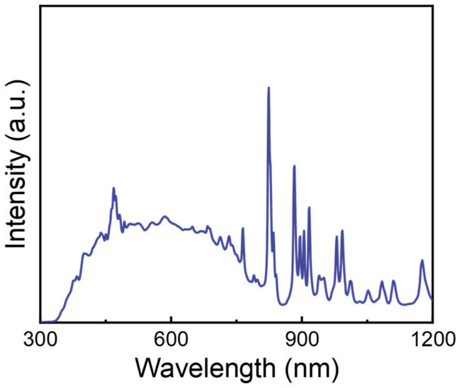

Supplementary Fig. 51. Emission spectrum of the xenon-lamp solar simulator used for the stability measurements. The spectrum was obtained from the manufacturer-provided spectral report for the xenon lamp light source configuration used in this work.

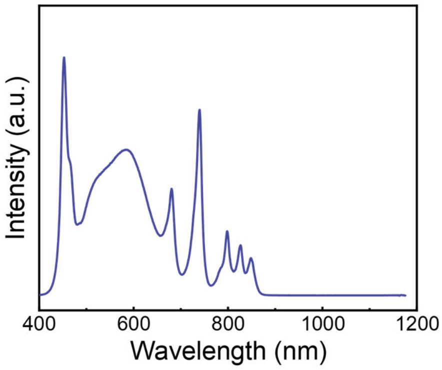

Supplementary Fig. 52. Emission spectrum of the white-light LED source used for stability measurements. The spectrum was obtained from the manufacturer-provided spectral report for the LED light source configuration used in this work. The y-axis represents the relative spectral intensity (arbitrary units).

Supplementary Tables

Supplementary Table 1. Grain Boundary Energy (J/m^2)

Grain boundary types	Σ3(111)	Σ5(130)	Σ5(120)	Σ3(112)
Grain boundary energy (J/m^2)	0.0340	0.0163	0.0171	0.0356

Supplementary Table 2. TRPL fitted parameters were obtained from a bi-exponential fitting equation.

Samples	τ_1 (ns)	A_1 (%)	τ_2 (ns)	A_2 (%)	τ_{ave} (ns)
Control	101.00	87.79	1412.10	12.21	966.83
Target	538.20	43.34	3884.70	56.66	3564.04

Supplementary Reference

1. Dokic, J. et al. Quantum chemical investigation of thermal cis-to-trans isomerization of azobenzene derivatives: substituent effects, solvent effects, and comparison to experimental data. *J. Phys. Chem. A*, **113**, 6763–6773 (2009).
