# Supplementary material for: Photoswitchable isomers to improve grain boundary resilience and perovskite solar cells stability under light cycling
Source: Nat Energy. 2026 Feb 25;11(4):623–32. doi: 10.1038/s41560-026-01993-z (PMC13121018; doi:10.1038/s41560-026-01993-z)
Supplement: Supplementary file 2 — Reporting Summary [file 41560_2026_1993_MOESM2_ESM.pdf]

nature portfolio

Corresponding author(s): Guixiang Li, Luyao Wang, Antonio Abate, Michael Saliba, Meng Li

Solar Cells Reporting Summary

Nature Portfolio wishes to improve the reproducibility of the work that we publish. This form is intended for publication with all accepted papers reporting the characterization of photovoltaic devices and provides structure for consistency and transparency in reporting. Some list items might not apply to an individual manuscript, but all fields must be completed for clarity.

For further information on Nature Research policies, including our data availability policy, see Authors & Referees.

Experimental design

Please check the following details are reported in the manuscript, and provide a brief description or explanation where applicable.

1. Dimensions

Area of the tested solar cells
- [x] Yes — The area of the tested solar cells is 0.18 cm2.
- [ ] No — *Explain why this information is not reported/not relevant.*

Method used to determine the device area
- [x] Yes — The active area was determined by the aperture shade ma placed in front of the solar cell.
- [ ] No — *Explain why this information is not reported/not relevant.*

2. Current-voltage characterization

Current density-voltage (J-V) plots in both forward and backward direction
- [x] Yes — Provided in Supplementary Fig. 42.
- [ ] No

Voltage scan conditions
- [x] Yes — The J–V curves were recorded with a black mask with an active area of 0.0982 cm2 using a Keithley 2400 SourceMeter and a solar simulator under AM 1.5G illumination. The light intensity was calibrated through a reference silicon solar cell obtained from National Institute of Standards and Technology (America). are forward (from -0.1 V to 1.22 V) and reverse (from 1.22 to -0.1 V) scan with a speed of 20 mV/s and dwell time of 10 ms.
- [ ] No — *Explain why this information is not reported/not relevant.*

Test environment
- [x] Yes — The J-V plots are tested in the N2 glove box at room temperature with relative humidity below 20%.
- [ ] No — *Explain why this information is not reported/not relevant.*

Protocol for preconditioning of the device before its characterization
- [ ] Yes — *Provide a description of the protocol.*
- [x] No — No preconditioning is needed.

Stability of the J-V characteristic
- [x] Yes — Supplementary Fig. 43: Sample temperature: room temperature, irradiance: 1000 W m-2, continuously scan the maximum power point of sample for 400 seconds. Take the average Pmax. The stabilized average PCE of 27.0% determined from MPPT for 400 s.
- [ ] No — *Explain why this information is not reported/not relevant.*

3. Hysteresis or any other unusual behaviour

Description of the unusual behaviour observed during the characterization
- [ ] Yes — *Provide a description of hysteresis or any other unusual behaviour observed during the characterization.*
- [x] No — low hysteresis was observed for target devices.

Related experimental data
- [x] Yes — J-V curves under reverse and forward scans were provided in Supplementary Fig. 42. For the control devices, the power conversion efficiencies (PCEs) under forward and reverse scans are 24.5% and 25.6%, respectively. For the target devices, the corresponding PCEs are 26.9% and 27.2%, respectively.
- [ ] No — *Explain why this information is not reported/not relevant.*

4. Efficiency

External quantum efficiency (EQE) or incident photons to current efficiency (IPCE)

☒ Yes
☐ No

Supplementary Fig. 44. The integrated JSC of the champion inverted PSC form EQE curve is 25.78 (control) and 26.15 (target) mA cm-2.

Explain why this information is not reported/not relevant.

A comparison between the integrated response under the standard reference spectrum and the response measure under the simulator

☒ Yes
☐ No

The integrated JSC values obtained from EQE were agree well with the JSC determined from J-V curve.

Explain why this information is not reported/not relevant.

For tandem solar cells, the bias illumination and bias voltage used for each subcell

☐ Yes
☒ No

Provide a description of the measurement conditions.

No tandem solar cells were reported in our manuscript.

5. Calibration

Light source and reference cell or sensor used for the characterization

☒ Yes
☐ No

A solar simulator (EnliTech, SS-X50, with A+ spectrum) and a silicon solar cell obtained from National Institute of Standards and Technology (America).

Explain why this information is not reported/not relevant.

Confirmation that the reference cell was calibrated and certified

☒ Yes
☐ No

The light intensity was calibrated through a reference silicon solar cell obtained from National Institute of Standards and Technology (America).

Explain why this information is not reported/not relevant.

Calculation of spectral mismatch between the reference cell and the devices under test

☐ Yes
☒ No

Provide a value of the spectral mismatch and/or a description of how it has been taken into account in the measurements.

We rely on certified efficiency results.

6. Mask/aperture

Size of the mask/aperture used during testing

☒ Yes
☐ No

Metal aperture masks with areas of 0.0982 and 0.0782 cm2 were used for testing.

Explain why this information is not reported/not relevant.

Variation of the measured short-circuit current density with the mask/aperture area

☐ Yes
☒ No

Report the difference in the short-circuit current density values measured with the mask and aperture area.

All J-V curves were measured with a metal aperture mask.

7. Performance certification

Identity of the independent certification laboratory that confirmed the photovoltaic performance

☒ Yes
☐ No

The National PV Industry Measurement and Testing Center.

Explain why this information is not reported/not relevant.

A copy of any certificate(s)

☒ Yes
☐ No

Supplementary Figs. 46 and 47.

Explain why this information is not reported/not relevant.

8. Statistics

Number of solar cells tested

☒ Yes
☐ No

Fig. 3c and Supplementary Fig. 41: collected from 16 individual cells for each condition.

Explain why this information is not reported/not relevant.

Statistical analysis of the device performance

☒ Yes
☐ No

Provide in Fig. 3c and Supplementary Fig. 41.

Explain why this information is not reported/not relevant.

9. Long-term stability analysis

Type of analysis, bias conditions and environmental conditions

☒ Yes
☐ No

Light stability of unencapsulated devices under continuous 1-sun-equivalent white-light LED illumination at 65 °C with maximum power point tracking in N2 (Fig. 4a). The light cycling stability testing of 12 hours light and 12 hours dark under LED and xenon light in an N2 atmosphere at and 65°C (Fig. 4b and c). The temperature cycling stability testing of -40-85°C under xenon light in a vacuum environment (Fig. 4d).

Explain why this information is not reported/not relevant.
